# Multifunctional, energy-autonomous textile sensors enabled by spray-coated two-dimensional heterostructures

**DOI:** 10.1038/s41528-026-00539-3

**Published:** 2026-02-24

**Authors:** Evgeniya Kovalska, Jack Routledge, Rocco Cancelliere, Hoi Tung Lam, Kavya Sreeja Sadanandan, Bing Wu, Liping Liao, Zdenek Sofer, Ana I. S. Neves, Saverio Russo, Laura Micheli, Monica F. Craciun

**Affiliations:** 1 Department of Engineering, Faculty of Environment, Science and Economy, University of Exeter, Exeter, UK; 2 Department of Physics and Astronomy, Faculty of Environment, Science and Economy, University of Exeter, Exeter, UK; 3 Department of Chemical Sciences and Technologies, University of Rome Tor Vergata, Via della Ricerca Scientifica 1, Roma, Italy; 4 Department of Inorganic Chemistry, University of Chemistry and Technology Prague, Prague, Czechia

**Keywords:** Energy science and technology, Engineering, Materials science, Nanoscience and technology

## Abstract

Two-dimensional (2D) materials offer unprecedented opportunities for energy-autonomous wearable electronics, yet their scalable and environmentally friendly integration into textiles remains a major challenge. Here, we introduce an ultrasonic spray-coating method to fabricate water-processable, surfactant-free 2D heterostructures comprising graphene and transition metal dichalcogenides (TMDs) as electronic dyes on textile fabrics. The resulting lightweight (~1 g/device), flexible textile-integrated triboelectric nanogenerators (TENGs) demonstrate a record-high power density of 793 mW m^-2^ among single-phase TMD-based textile devices. These TENGs enable self-powered, wearable detection of environmental and physiological parameters, including atmospheric humidity, body temperature, and volatile organic compounds (VOCs) such as acetone and styrene, via a tap-to-sense mechanism. The sensor achieves a record-breaking responsivity of 126% for styrene vapours, making it the first wearable, self-powered styrene sensor. The device’s multifunctionality – driven by thermal modulation of charge transport in the MoS_2_ layer – enables reliable body temperature detection with minimal cross-sensitivity to humidity or VOCs, crucial under real-world fluctuations. The sensor maintains mechanical resilience and operational stability over 80 days of continuous use and after 200 bending cycles. This work advances scalable, sustainable strategies for multifunctional, self-powered textile sensors and paves the way toward wearable personalised healthcare technologies with accurate multiparameter sensing.

## Introduction

Current portable medical-grade sensing technologies are heavily reliant on external power sources, such as electrical mains or batteries, which limits their functionality and usability. In contrast, self-powered wearable sensors integrated into textiles present transformative opportunities for personalised healthcare solutions, enabling targeted medical interventions at the point of care^1,2^. By integrating energy harvesting technologies that draw power from ambient sources like light, motion, or heat, self-powered medical devices are paving the way for more sustainable, patient-centred healthcare solutions^3^. Self-powered systems leveraging triboelectric nanogenerator (TENG) technologies offer a transformative opportunity to develop energy-autonomous healthcare sensing platforms that harness energy from the ubiquitous source of human (bio)mechanics^4^. The voltage or current outputs of the TENG can serve as sensing signals that reflect mechanical, chemical, or other stimuli applied to the device. Owing to the high sensitivity of triboelectrification to charge transfer between contact materials, advanced material design holds the potential to drive a paradigm shift in sensing, integrating exceptional sensitivity and selectivity with energy-autonomous operation^5^. Furthermore, embedding this technology directly into textiles can significantly enhance patient compliance, enabling discreet health monitoring, particularly for individuals who struggle with bulky and intrusive devices, such as those with neurodegenerative diseases^6^.

Layered two-dimensional (2D) materials are redefining textile-based wearable technologies through their exceptional mechanical flexibility, tuneable electronic properties, and seamless integration into multifunctional, high-sensitivity biosensing platforms^7,8^. These materials have enabled the development of a wide range of powered healthcare sensors^7–9^, facilitating monitoring of volatile organic compounds (VOCs)^10–14^, vital human signs such as respiration and pulse^15^, human motion^16–18^, cardiac arrhythmia^19^, as well as a variety of other physiological signals including brain activity and muscle contractions^20^, along with indicators like body temperature^20–24^ and hydration levels^20^. While various 2D materials possess promising triboelectric properties^25^, most self-powered sensors based on 2D material-based TENGs are fabricated on plastic substrates, where performance is often restricted by material incompatibility, inefficient device architectures, and fabrication-induced interfacial losses. As a result, textile-integrated implementations therefore remain limited, typically employing 2D materials as electrodes^18,26,27^ or embedded in composite matrices such as polydimethylsiloxane (PDMS)^28^, polyvinyl alcohol (PVA)^29^, polyvinyl chloride (PVC)^30^, and polyaniline (PANI)^31^. These TENGs were demonstrated exclusively for human motion^18,28–32^, temperature^33^, and breathing rate^34^ sensing, or serving merely as energy sources to power commercial sensors^34,35^. Establishing a scalable and sustainable approach for fabricating textile-integrated self-powered TENG sensors that leverage 2D materials to enable medically relevant (bio)mechanical monitoring and detection of environmental and human physiological signals would have transformative implications beyond healthcare.

In this Article, we present a textile-based analogue of 2D heterostructures (2Dh) for a self-powered sensing wearable platform capable of continuous monitoring of environmental and human physiological signals – including humidity, body temperature, and VOCs – with applications in early disease detection. This functionality is enabled through the development of textile-based TENGs incorporating 2Dh systems composed of multilayer graphene (MLG) electrodes and transition metal dichalcogenides (TMDs), including MoS_2_, MoSe_2_ and WS_2_ as triboelectric layers, reaching a record-high power density of 793 mW m^-2^ among single-phase TMD textile devices. Our sustainable, solution-processed fabrication employs an isopropanol–water mixture to formulate stable, water-based 2D material dispersions used as electronic dyes, which are directly deposited onto polyester textiles via ultrasonic spray coating. This technique enables precise, layer-by-layer assembly of 2D heterostructures with micron-scale control and exceptional conformality on complex fibre geometries, offering unparalleled engineering of hierarchical 2Dh architectures on flexible textile substrates. By integrating TMD/MLG combinations, we merge graphene’s high electrical conductivity and mechanical durability with the triboelectric and chemoresponsive functions of TMDs to yield synergistic triboelectric output and multi-modal sensing capabilities. The resulting 2Dh-functionalised textiles demonstrate record-breaking responsivity, including a 126% response to styrene vapours, while exhibiting long-term operational stability, mechanical resilience under bending, and environmental robustness under high humidity conditions. Functionalising the MoS_2_ triboelectric layer with polythiophene nanoparticles further enhances selectivity and sensitivity to styrene, a key biomarker for Parkinson’s disease, significantly expanding the device’s diagnostic potential. Additionally, leveraging MoS_2_’s temperature-dependent electronic properties, the platform enables tap-to-sense detection of elevated skin or body temperature with minimal cross-sensitivity, critical for real-world applications where environmental factors vary simultaneously. Wireless data transmission integration completes a seamless, autonomous sensing system. This study establishes a new paradigm in self-powered wearable electronics by uniting triboelectric energy harvesting with solution-processed, electronically active 2Dh architectures directly embedded in fabrics. By combining sustainable synthesis, scalable ultrasonic heterostructure printing, and advanced materials functionalisation, our platform offers a powerful, autonomous route to continuous physiological and environmental monitoring, advancing the frontiers of personalised health diagnostics and smart textiles.

## Results

### 2D materials heterostructures as textile electronic dyes

**Fig. 1:**
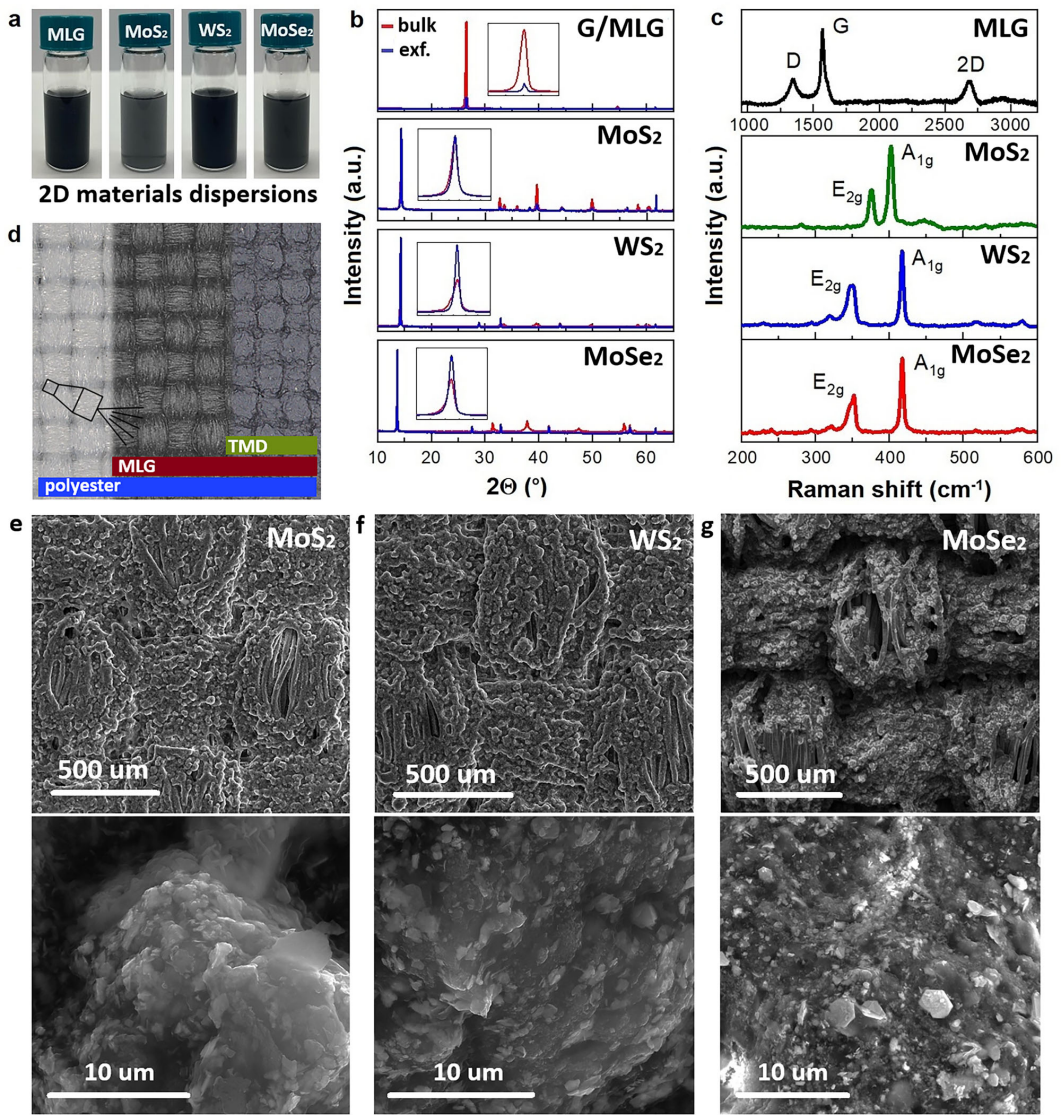
2D materials heterostructures deposition on textiles and their characterisation. **a** Digital photos of dispersions of MLG, MoS_2_, WS_2_, and MoSe_2_ in the mixture of DI (3 wt.%) and IPA (1 wt.%) **b** XRD patterns of the bulk and exfoliated materials; the insets display enlarged peaks corresponding to the (002) plane. **c** Raman spectra of the exfoliated 2D derivatives. **d** 2D optical image of polyester spray-coated with MLG and TMD (specifically MoS_2_), along with its schematic illustration, where blue represents polyester, maroon represents MLG and lime-green indicates TMD. SEM images of (**e**) MoS_2_, (**f**) WS_2_ and (**g**) MoSe_2_ on MLG deposited on textile are shown at different magnifications.

2D materials dyes (Fig. 1a) were prepared as stable dispersions by shear force exfoliation (see “Methods”) of commercial expanded graphite and bulk powders of MoS_2_, MoSe_2_, and WS_2_ in the mixture of isopropanol (IPA) and deionised water (DI) at the ratio 1:3, respectively. The exfoliation process took place for 2 h at a 7000 rpm rotation speed and was followed by decantation of the exfoliated materials after 30 minutes of sedimentation. The concentration of MLG, MoS_2_, WS_2,_ and MoSe_2_ in dyes was estimated as follows 1.5 gL^−1^, 1.9 gL^−1^, 1.7 gL^−1^, 1.2 gL^−1^. Figures S1–S5 show the measured particle diameter distribution with dynamic laser scattering (DLS) of the dispersions in a glass cuvette. These measurements reveal an average flake diameter of 260.8 nm (MLG), 231.4 nm (MoS_2_), 210.5 nm (WS_2_), and 150.3 nm (MoSe_2_). Atomic force microscopy (AFM) of as-exfoliated 2D materials reveals flakes with thicknesses between 20 and 60 nm, corresponding to 12–38 layers^36^ (Fig. S6). The structure of bulk and exfoliated materials was examined by X-ray diffraction (XRD, Fig. 1b), confirming that 2D materials in the dispersions have retained the 2H phase, i.e., indexed as hexagonal (*P63/mmc* space group) as expected^37,38^ for MoS_2_, WS_2,_ and MoSe_2_ with semiconducting properties^39^. Figure 1c shows the Raman spectra for each 2D material, with characteristic peaks corresponding to the dominant inelastic scattering processes. For MLG, the D, G, and 2D bands are found at 1342.2, 1568.1, and 2686.8 cm^−1^, respectively, as expected^40^. Raman spectra of exfoliated TMDs align with the literature^41–43^ and were observed at 376.1 and 402.3 cm^−1^ for MoS_2_, at 349.5 and 417.2 cm^−1^ for WS_2,_ and 241.4, 294.6 and 352.6 cm^−1^ for MoSe_2_.

The 2D material dyes were spray-coated on the textile utilising a SONO-TEK ExactaCoat spray coater (https://www.sono-tek.com/). The spray coater is equipped with an ultrasonic nozzle that prevents 2D particles’ precipitation and facilitates their uniform deposition on the textile substrate through controlled nozzle movement and the environment of coating conditions. Ultrasonic atomisation produces micron-scale droplets with a narrow size distribution, ensuring conformal and layer-by-layer deposition even on the complex fibre topography of woven textiles. This deposition method has proven especially effective for 2D heterostructure fabrication. The sequential coating of graphene followed by TMD layers enables precise spatial control over each constituent material while preserving the mechanical softness and flexibility of the underlying textile. Compared to other methods such as dip-coating or blade-casting, ultrasonic spray coating offers superior uniformity, reduced material waste, and compatibility with roll-to-roll manufacturing processes. Figure 1d shows an optical micrograph and corresponding schematic of a polyester textile (white/blue) coated with a 45–65 μm thick MLG electrode (black/maroon) obtained after 200 coating passes. This is followed by the deposition of 20–30 μm thick TMDs (MoS_2_, MoSe_2_, and WS_2_; dark grey/lime-green) as a triboelectric layer, for which 100 coating passes were conducted.

The morphology of sprayed 2Dh on polyester was evaluated by scanning electron microscopy (SEM; Figures 1e–g and S7). The low magnification SEM images (500 µm scale) reveal a uniform and conformal coating on the polyester with visible evidence of the textile’s yarn alignment and texture. At the same time, the high magnification SEM images (10 µm scale) enable a detailed examination of particle distribution with lateral sizes ranging from 200 nm up to 5 µm for all TMDs deposited on the polyester textile. The thickness of the 2Dh coatings was analysed by a 3D light microscope (Figs. S8–S10), indicating a deposition thickness of approximately 60–90 μm. The thickness of the MLG layer was previously optimised as an electrode for textile-based TENGs^27^, while the TMD layer thickness has also been recently refined for enhanced triboelectric performance^44^.

### 2D heterostructures-based TENG on textile

**Fig. 2:**
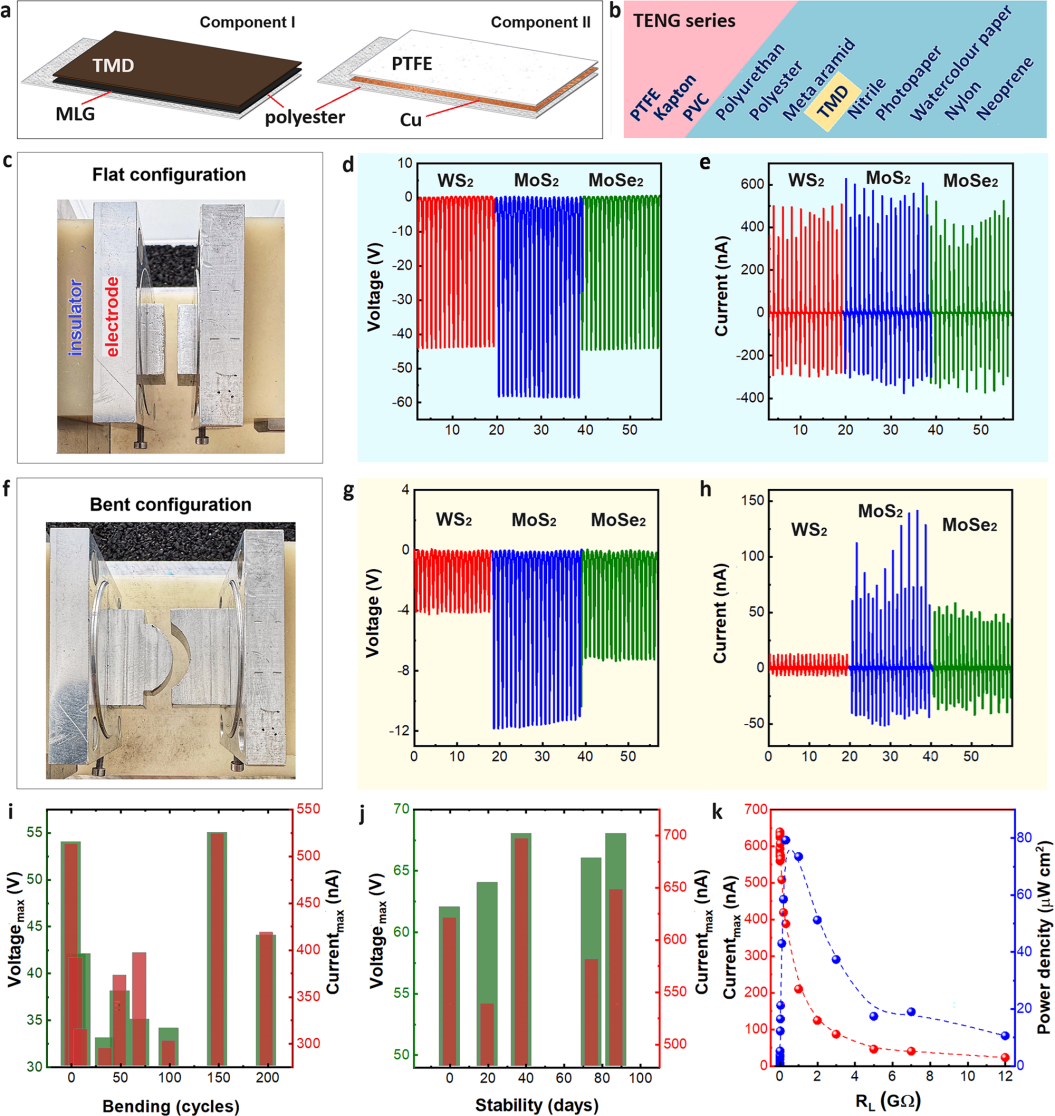
TMD/MLG-TENG device configuration and characterisation. **a** Schematic illustration of the TMD/MLG-TENG design. **b** TENG series of the tested triboelectric materials and the TMDs position in the series. **c** Digital photo of TMD/MLG-TENG in a flat configuration and its electrical output performance: **d** open circuit voltage, *V*_*oc*,_ and (**e**) short circuit current, *I*_*sc*_. **f** Digital photo of TMD/MLG-TENG in a bending configuration and its electrical output performance: **g** open circuit voltage and **h** short circuit current. **i** The open circuit voltage and short circuit current of MoS_2_/MLG-TENG over 200 bending cycles, and **j** over time. **k** Dependence of the maximum short-circuit current and power density of the MoS_2_/MLG-TENG on the resistance of the external load (5 kΩ–12 GΩ).

To assess the triboelectric performance of as-manufactured 2Dh on polyester, we designed a TENG in a double electrode configuration implemented in contact-separation (CS) mode as shown in Fig. 2a. The TENG device consists of two components. The first component is based on the 2Dh, where MLG is sprayed onto a polyester fabric substrate, and the TMD (MoS_2_, MoSe_2_, or WS_2_) is layered on top of the MLG. This configuration creates a durable electron-donor triboelectric layer. The second component consists of Cu on a polyester fabric substrate, with a layer of polytetrafluoroethylene (PTFE) on top of the Cu electrode. The PTFE serves as the triboelectric pair to TMD, possessing electron-acceptor properties. This configuration facilitates charge transfer in alignment with the triboelectric series, as shown in Figs. 2b and S11. The device dimensions are 1.5 cm × 2.5 cm, and the total weight is ~1 g/device, making it exceptionally compact and lightweight. This small form factor facilitates seamless integration into wearable systems, conformable electronics, and other space-constrained platforms, while the low mass ensures minimal mechanical burden for applications requiring flexibility, portability, or skin attachment. The dimensional efficiency is also critical for areas where device miniaturisation and weight reduction are essential for user comfort, mobility, and unobtrusive operation.

Distinct TMD/MLG-TENG devices were fabricated, based on various TMD, i.e., MoS_2_, WS_2_ and MoSe_2_^26^, and their energy harvesting performance was characterised in the flat configuration as shown in Fig. 2-c. The measurements of electrical output in ambient conditions were performed in contact-separation (CS) mode at a frequency of 1 Hz (see “Methods”). Figures 2d, e and S12a show that the highest open circuit voltage output (*V*_*oc*_) is generated by the MoS_2_/MLG-TENG, reaching ~60 V, with WS_2_ and MoSe_2_ producing outputs of ~45 V. The short circuit currents (*I*_*sc*_) produced by the devices reach ~600 nA for MoS_2_, and ~450 nA for WS_2_ and MoSe_2_. Finally, the highest charge accumulation (*Q*_*oc*_) value of 23 nC was measured in MoS_2_-based devices, while 15 nC was observed for WS_2_/MLG-TENG and MoSe_2_/MLG-TENG, respectively. These variations in output across different TMD materials occurred despite similar surface roughness and flake morphology, as confirmed by morphological analysis in Fig. 1. This suggests that the performance differences stem from the intrinsic electronic properties of the TMDs rather than morphological factors. Specifically, MoS_2_ may exhibit a more favorable electron-donating character in contact with PTFE, leading to greater triboelectric charge generation. Additionally, improved interfacial charge transfer at the MoS_2_/MLG junction, due to better band alignment or lower contact resistance, may contribute to the observed enhancement in electrical output. Similar measurements were performed for the aforementioned TMD/MLG-TENG devices to assess their performance during bending (Fig. 2f and S12b), indicating a greater electrical output for MoS_2_/MLG-TENG in bent configuration compared to other samples, albeit with a lower performance than in the flat configuration (Figs. 2g, h). All devices showed a similar decrease in performance under bending, regardless of the TMD used, indicating that mechanical deformation affects all heterostructures in a comparable way, likely due to strain-induced changes in contact area or interfacial integrity and local rearrangement within interconnected 2D flake networks bridging textile fibres. The consistent bending response, combined with the clear material-dependent output in flat conditions, highlights the critical role of TMD selection in optimising TENG performance for wearable or flexible applications.

Considering the best electrical capabilities of MoS_2_/MLG-TENG, we conducted device evaluations to explore its efficiency, durability and potential applications. To demonstrate the feasibility of MoS_2_/MLG-TENG for wearable electronics, we analysed its flexibility and durability through 200 bending cycles. The device reveals relatively stable performance with an average electrical output of ~40 V, 400 nA, and 15 nC (Figs. 2i and S13a). Minor changes were attributed to the textile’s non-uniform structure and realignment of 2D material flakes–the non-immediate reassembling of flakes to the same position after each bending cycle contributed to these variations. Consistent electrical performance after 200 bending cycles highlights the mechanical robustness of the flake-based heterostructure and aligns with previous textile-based TENG studies^27^, where ultrasonically spray-coated 2D networks exhibited mechanically robust behaviour, emphasising the device’s potential for wearable applications.

The MoS_2_/MLG-TENG’s performance was evaluated over time, revealing notable stability over a period of 80 days. Insignificant differences in electrical output (~5 V, 150 nA and 5 nC) were observed (Fig. 2j and S13b) and attributed to variations in ambient conditions in the room, such as humidity and temperature.

To quantify the power density of the MoS_2_/MLG-TENG, we analysed the device’s output current (*I)* across various load resistances ranging from 5 kΩ to 12 GΩ, revealing a gradual decay as the load resistance (*R)* increased (Fig. 2k). The output power density (*P*_*d*_) exhibited an increase, reaching its maximum at 79.3 µW cm^−2^ (or 793 mW m^−2^) under a 300 MΩ load, which corresponds to the effective impedance matching condition of the device under the applied mechanical excitation. The calculation of *P*_*d*_ utilised the formula: *P*_*d*_ = *I*^*2*^*·R·A*^*-1*^, where *A* is the active area of the device.

While TMD-based textile-integrated TENGs are emerging as a rapidly advancing frontier within energy-autonomous systems, most studies to date have focused on incorporating TMDs into composite matrices such as PDMS^28^, PVA^29^, or PVC^30^, primarily for energy harvesting applications designed to power external sensor devices. In contrast, this work advances a new direction in energy autonomy by demonstrating self-powered sensing, where the same textile-integrated TENG both harvests energy and performs detection. Critically, we employ single-phase TMDs directly deposited onto textile substrates, an essential design choice for sensing applications, where the active material must remain fully accessible for analyte interaction. Composite embedding, while suitable for mechanical energy harvesting, can hinder this accessibility and limit multifunctional sensing capabilities. The MoS_2_/MLG-TENG developed in this study overcomes these constraints, achieving a record-high power density among textile-integrated devices based on single-phase TMDs, as detailed in Table 1, which benchmarks performance across the current literature.

**Table 1:** Comparative analysis of MLG/MoS_2_ TENG output parameters with recent TMD-based textile-integrated TENG devices used for sensing applications

Device structure Electrode1/ Tribolayer1: Tribolayer2/ Electrode2	2D material integration	Textile	*V*_*oc*_ V	*I*_*sc*_ density μA cm^−2^	*P*_*d*_ mW m^-2^	Application	Year. [ref]
**WS**_**2**_:PTFE/Al	WS_2_ drop casted on the textile	Silk textile	7	0.17	2.7	Motion sensor	^32^
Cu/**PP-Ti-MoS**_**2**_ : Nylon /Ag	MoS₂ doped with titanium, drop-casted on polypropylene (PP) cloth	PP cloth	10	15	−	Breathing rate sensor	^34^
**CC@MoS**_**2**_ : Ta_4_C_3_	Hydrothermal growth of MoS_2_ on carbon cloth (CC)	Carbon cloth	0.88	15.68	39	Running speed sensor. Wireless warning system	^35^
Cu/Cotton-**AuWS**_**2**_ **:**PTFE	Au-doped WS_2_ drop-casted on textile	Cotton fabric	11.6	0.06	1.06	Temperature sensor	^33^
Graphene/**MoS**_**2**_ : PTFE/Cu	Ultrasonic spray coating of graphene and MoS_2_ layered structure	Polyester fabric	65	0.24	793	Multifunctional sensor: humidity, VOC, temperature	This work

Most wearable sensing devices reported to date have been designed to monitor a single parameter, typically human motion, temperature, or breathing rate. However, practical applications in personalised health monitoring and environmental diagnostics demand sensors capable of detecting multiple parameters simultaneously, in order to reduce system complexity, volume, weight, and cost. A compelling solution to this challenge is the development of a multifunctional sensing platform that integrates diverse sensing modalities within a single architecture. In the following sections, we demonstrate that our MoS_2_/MLG-TENG operates as a multifunctional sensor, capable of simultaneously detecting humidity, temperature, and a range of VOCs, thereby offering a compact and efficient approach for comprehensive physiological and environmental monitoring.

### Self-powered, triboelectric-driven wearable sensors

**Fig. 3:**
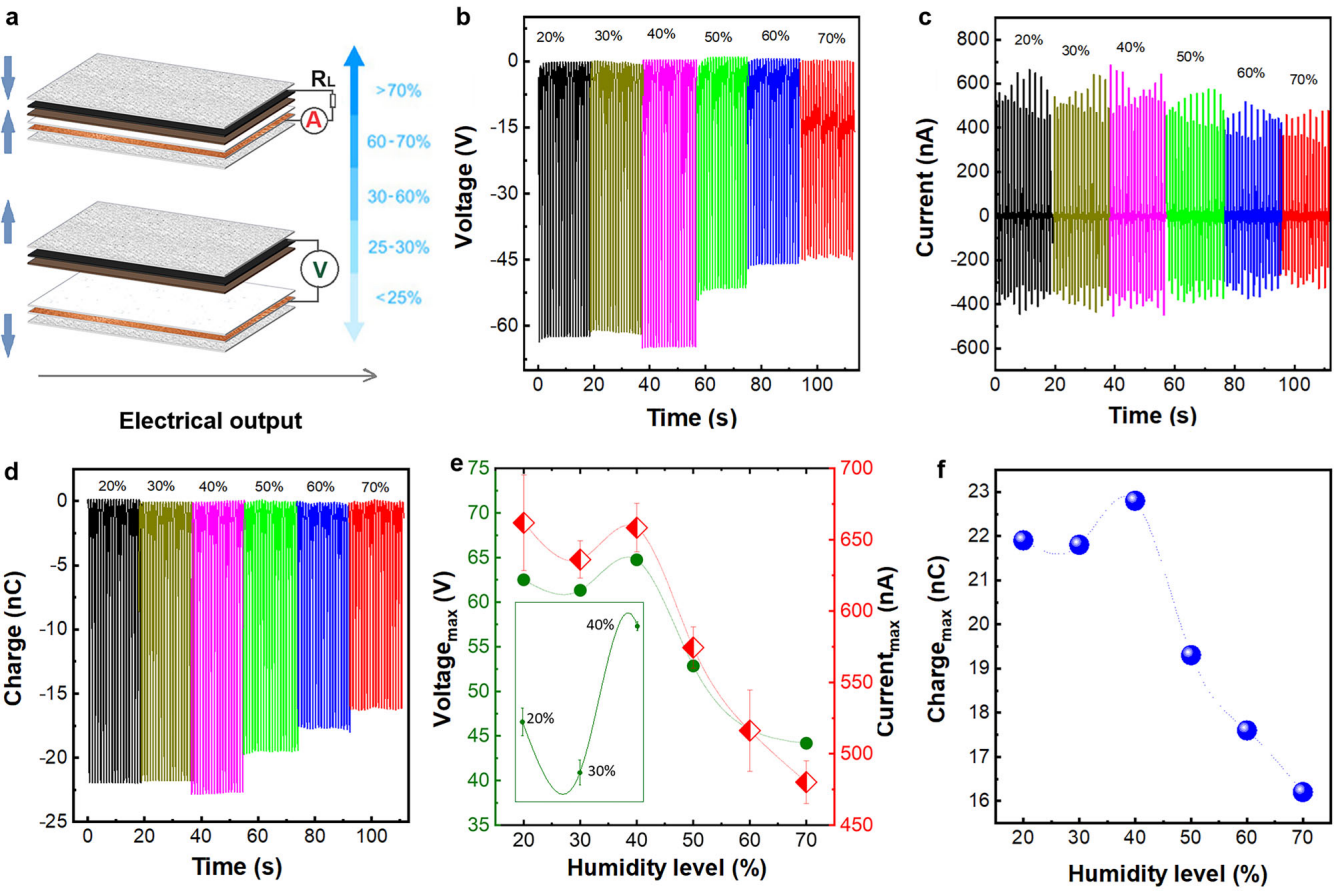
MoS_2_/MLG-TENG enabled humidity sensor. **a** Schematic illustration of the device’s performance evaluation in contact-separation mode under different humidity conditions. **b** Open-circuit voltage, **c** short-circuit current and **d** open-circuit charge of the device measured at 20–70% humidity as a function of time. **e** Maximum open-circuit voltage and short-circuit current as a function of relative humidity. Data points represent the average value over all measured voltage peaks for each humidity level. Error bars for both voltage and current correspond to the standard deviation from the mean, calculated from the distribution of the measured voltage peaks at each humidity level. The inset shows a magnified view of the voltage signal, for which the error bars are smaller than the data-point size.

To assess the suitability of the MoS_2_/MLG-TENG for wearable applications across diverse environments, we first evaluated the device in CS mode under varying relative humidity (RH) conditions (Fig. 3a), ranging from 20% to 70%. Figures 3b–d demonstrate the device’s significant stability within the 20% to 40% RH range, achieving the highest electrical outputs of ~65 V, 700 nA, and 23 nC as summarised in Figs. 3e, f. The humidity dependence of the maximum voltage, current, and transferred charge, defined as the average of the peak maxima measured at each humidity level, is summarised in Fig. 3e, f, where error bars represent the standard deviation from the mean. A 15–31% decrease in electrical output was observed when the device operated in higher humidity (50–70% RH), likely due to the adsorption and desorption of water molecules on the surface defects of MoS_2_. Notably, the device’s ability to restore its performance suggests that the (ad)desorption processes are reversible, indicating the absence of saturation effects at the edges of MoS_2_ structures^45^.

Beyond steady-state operation, the dynamic humidity response of the device was further investigated through the evaluation of key performance metrics, including response time, recovery time, and sensing resolution. During continuous operation of the TENG measurement, increasing the humidity induces a clear temporal shift in the voltage peaks (Figs. S14a, S14b), reflecting the sensor response to increasing moisture levels. The response time is defined as the time interval between consecutive voltage peaks immediately before and after the humidity increase and is determined to be Δ*T* ≈ 0.98 s. Upon subsequent reduction of humidity through argon purging, the voltage signal rapidly returns to its initial temporal position (Figs. S14c, S14d), indicating sensor recovery. The recovery time is defined analogously as the time interval between consecutive voltage peaks immediately before and after the decrease in humidity and is also found to be Δ*T* ≈ 0.98 s. Simultaneous measurements using a commercial humidity sensor (GoveeLife Thermometer Hygrometer; see Fig. S15) were employed to calibrate the applied humidity variations, which are consistent with the observed electrical response, demonstrating the rapid and reversible humidity sensitivity of the MoS_2_/MLG-TENG sensor. The humidity sensing resolution of the MoS_2_/MLG-TENG sensor (Fig. S16) was estimated to be on the order of 0.1% by correlating the smallest resolvable changes in the voltage response with the calibrated relative humidity variations measured using the commercial reference sensor (Fig. S15).

The self-powered humidity sensor response was quantified using the following normalised responsivity metrics *R*_*V*_*[%]=[(V*_*oc*_^*0*^*−V*_*oc*_^*s*^*)/V*_*oc*_^*0*^*]x100* and *R*_*I*_*[%]=[(I*_*sc*_^*0*^*−I*_*sc*_^*s*^*)/I*_*sc*_^*0*^*]x100*, which represent the relative change in *V*_*oc*_ and *I*_*sc*_ upon exposure to stimuli (in this case, humidity). This approach is analogous to response definitions in conventional humidity sensors, such as resistive or capacitive. Based on these, we obtain a voltage responsivity of *R*_*v*_ = 32% and a current responsivity *R*_*I*_ = 29% at 70% humidity. Collectively, these results showcase not only the device’s feasibility to withstand humid conditions, but also to act as a humidity sensor.

**Fig. 4:**
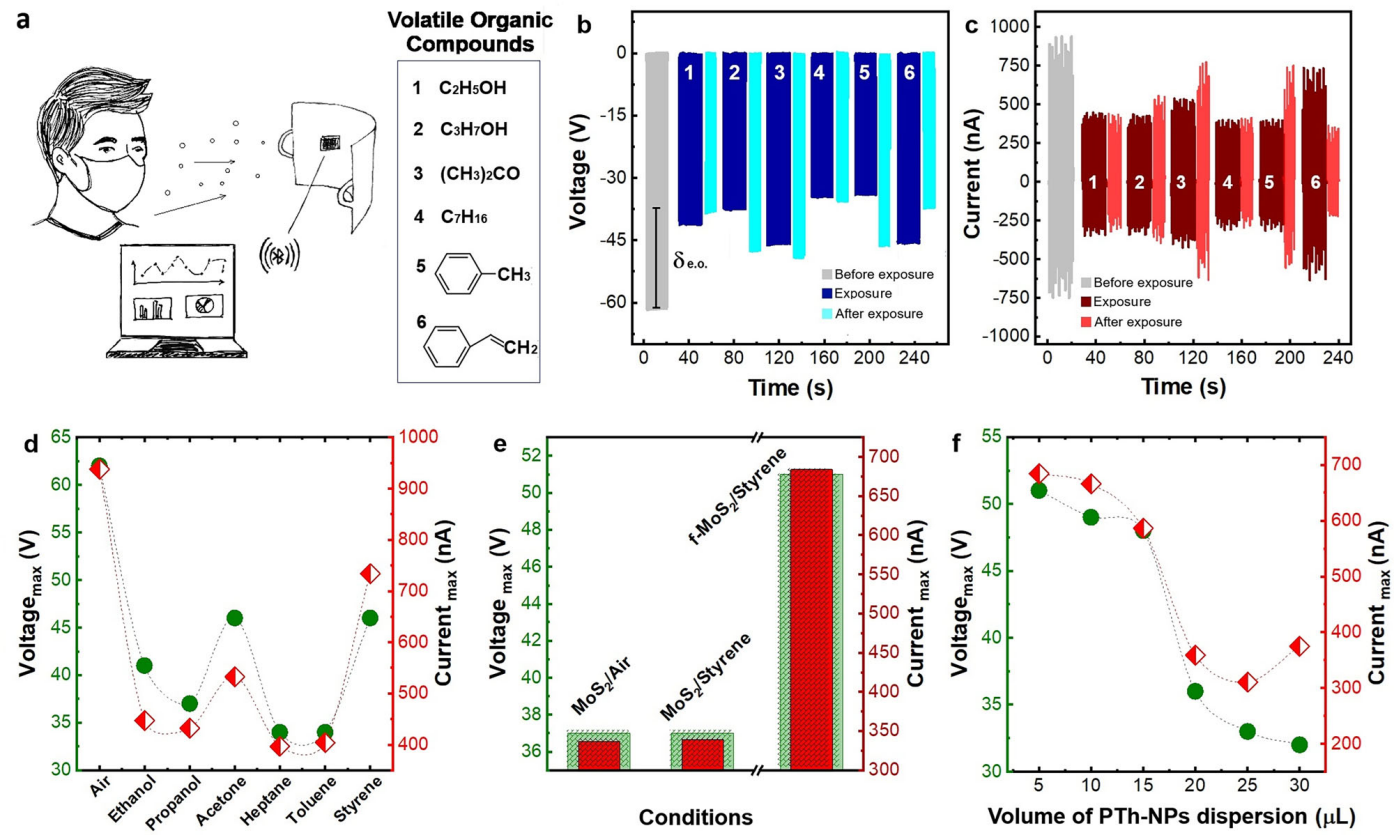
MoS_2_-TENG VOC vapour sensor. **a** Illustration of the wearable vapour sensor for breath detection and analysis of selected VOCs – ethanol (1), propanol (2), acetone (3), heptane (4), toluene (5) and styrene (6). **b** Electrical voltage output (*V*_*oc*_) under exposure to various VOCs; *d*_*e.o*._ indicates the difference in electrical output values measured in the initial conditions. **c** Current output (*I*_*sc*_) of the device under exposure to various VOCs. **d** Maximum output voltage and current vs. different analytes. **e** Comparison of the electrical output of the bare and functionalised device (*V*_*oc*_ and *I*_*sc*_) under exposure to styrene, and **f** as a function of a volume of 1 w/v % PTh-NPs dispersion in water.

Detecting VOCs in human breath or environmental settings is essential for both healthcare and safety^46–57^. Leveraging their intrinsic electrical properties and high surface sensitivity, 2D materials have emerged as promising candidates for VOC detection^12,48,49^, of particular interest for wearable systems, such as vapour sensors integrated into a face mask for breath analysis, as illustrated in Fig. 4a. Herein, we investigate the VOC sensitivity of MoS_2_/MLG-TENG by characterising its triboelectric performance (*V*_*oc*_, *I*_*sc*_, *Q*_*oc*_) under exposure to selected analytes. Tests were conducted in atmospheres (“exposure”) created by concentrated solutions of ethanol, propanol, acetone, heptane, toluene, and styrene, chosen due to their presence in human breath, particularly in individuals with conditions such as lung cancer, ketoacidosis, and Parkinson’s disease. The MoS_2_/MLG-TENG sensor’s output performance was lower under “exposure” compared to control conditions (“before exposure”, where the device consistently yielded outputs of ~900 nA, 62 V, and 21 nC), as indicated by the grey curves in Figs. 4b, c and S17, suggesting a reduction in efficiency post-exposure due to the adsorption of VOC molecules. Ultimately, as shown in Figs. 4d and S18, the sensor demonstrated a pronounced response to acetone vapours, generating outputs of ~500 nA, 46 V, and 16 nC. Similarly, it exhibited a notable response to styrene vapours, producing outputs of ~700 nA, 46 V, and 16 nC. Conversely, exposure to ethanol, propanol, and heptane vapours resulted in a significant decrease in electrical output, up to 50%. This behaviour is attributed to the nature of molecular interactions between acetone or styrene and the MoS_2_ triboelectric layer. While alcohols such as ethanol and propanol are more polar due to their hydroxyl (–OH) groups, these groups form strong intermolecular interactions, such as hydrogen bonding, which dominate over the weaker London dispersion forces present in molecules like acetone and styrene. These strong interactions can reduce the mobility of surface charges and hinder effective charge transfer, leading to lower triboelectric output. In contrast, the MoS_2_ triboelectric layer exhibits a greater surface affinity for acetone and styrene molecules, likely due to their specific functional groups (such as alkyl, CH_3_–, and vinyl, CH_2_ = CH–) and their significant positive inductive effects that enhance charge accumulation. This stronger charge induction compensates for their lower overall polarity, resulting in a higher electrical output compared to alcohols. Despite the strong sensor response post-exposure, the device requires additional time to fully recover “after exposure” as VOC molecules temporarily interfere with triboelectric performance. In this configuration, the MoS_2_ triboelectric layer necessitates extended VOC desorption to restore its surface properties.

Furthermore, we investigated the sensitivity of our sensor to styrene, which is not only classified as a hazardous VOC but also recognised as a biomarker for Parkinson’s disease^50–52^. Recent studies have proposed the fluorometric quantification of styrene using polythiophene nanoparticles (PTh-NPs)^55^. These nanoparticles can detect the presence of styrene by enhancing fluorescence emission in both water and air conditions^56^. Leveraging this concept, we enhanced the styrene sensitivity performance of MoS_2_/MLG-TENG by functionalising the MoS_2_ layer with PTh-NPs (Figs. 4e and S19). Additionally, we modified the MoS_2_ layer with varying concentrations of PTh-NPs by drop-casting different quantities (ranging from 1 to 6 drops, 5 µL each), as shown in Figs. 4f and S20. Subsequently, saturated vapours of concentrated styrene solution were analysed in a sealed chamber. A significant enhancement in performance was observed when comparing the bare (MoS_2_/MLG-TENG) and the PTh-NPs-functionalised TENG (f-MoS_2_/MLG-TENG), confirming the suitability of PTh-NPs as a selective electrical probe for styrene. A non-linear relationship was observed between the amount of PTh-NPs and the response to styrene at saturation. This suggests a dual response mechanism^55^: at lower PTh-NPs concentrations, the increase in current is due to analyte interaction with polymer chains, while at higher concentrations, PTh-NPs form nano-dispersions, leading to a decrease in current. These results demonstrate the feasibility of using MoS_2_/MLG-TENG for styrene detection.

The values for the various voltage and current responsivities (defined analogously to the humidity responsivities) are reported in Table 2 and highlight the ability of the MoS_2_/MLG-TENG sensor to perform both multifunctional sensing and selective VOC detection. Different from humidity sensing, which shows different values for voltage and current responsivities (*R*_*v*_ = 32%, *R*_*I*_ = 29%), the VOC sensor displays compound-specific response profiles for diverse VOCs, enabling simultaneous multiparameter readout and analyte discrimination. Notably, styrene induces a distinctly high current responsivity (*R*_*I*_ = 126%) coupled with a voltage responsivity of 65%, whereas acetone presents a lower and more symmetric profile (*R*_*v*_ = 24%, *R*_*I*_ = 42%). Ethanol and propanol, although chemically related, yield diverging voltage responsivities with closely matched current outputs, allowing for differentiation based on dual-signal analysis. Similarly, heptane and toluene both show voltage responsivities of 48%, yet their distinct current responsivities support fine discrimination. These variations are not only distinct between analytes but also fundamentally different from the humidity response, reinforcing the sensor’s chemical selectivity. This multidimensional responsivity space effectively transforms the TENG into a self-powered electronic nose capable of VOC fingerprinting, without the need for additional selective coatings or signal conditioning circuits, opening pathways for its application in non-invasive diagnostics, environmental sensing, and wearable health monitoring.

**Table 2:** Comparative responsivities of MoS_2_/MLG-TENG for various VOCs investigated

VOC	Voltage responsivity (R_v_)	Current responsivity (R_I_)
acetone	24%	42%
ethanol	33%	52%
propanol	40%	53%
heptane	48%	58%
toluene	48%	56%
styrene	65%	126%

The self-powered TENG sensor presented in this work represents a significant breakthrough as the first wearable device of its kind capable of styrene detection without the need for an external power source. Leveraging a hybrid material system of multilayer graphene and MoS₂ integrated onto textile substrates, this sensor achieves an unprecedented responsivity of 126%, outperforming recently reported styrene sensors based on fluorescent materials^53–56^ and photoionisation detection methods^57^, which exhibit responsivities ranging from 27% to 78%. Moreover, the TENG sensor exhibits an immediate and reproducible electrical response upon exposure, without the need for external power or thermal activation. In contrast to many reported fluorescent VOC sensors that require prolonged equilibration times, the fully textile-integrated and wearable nature of the MoS_2_/MLG-TENG enables direct, on-body detection with rapid signal stabilisation. Table 3 summarises this comparative analysis, highlighting the unique combination of self-powered operation, high responsivity, and wearable integration.

**Table 3:** Comparative analysis of MoS_2_/MLG-TENG and recent styrene sensors

Sensor type	Material/ System	Detection method	Responsivity	Response time	Wearable	Year [ref]
Self-powered TENG	MLG/**MoS**_**2**_ :PTFE/Cu	Modulation of triboelectric output	126%	immediate	yes	This work
Fluorescent	Eu/Zr-UiO-66	Fluorescence quenching	78%	30 s	no	^53^
Fluorescent	Eu@TMA-ME/FTO	Fluorescence quenching	75%	40 s	no	^54^
Fluorescent	Polythiophene nanoparticles	Fluorescence quenching	30%	15 min	no	^55,56^
Photo ionisation detector (PID)	Fourier-transform infrared (FTIR) spectrometer coupled to a PID	FTIR Spectroscopy	27-30%	10 min	no	^57^

**Fig. 5:**
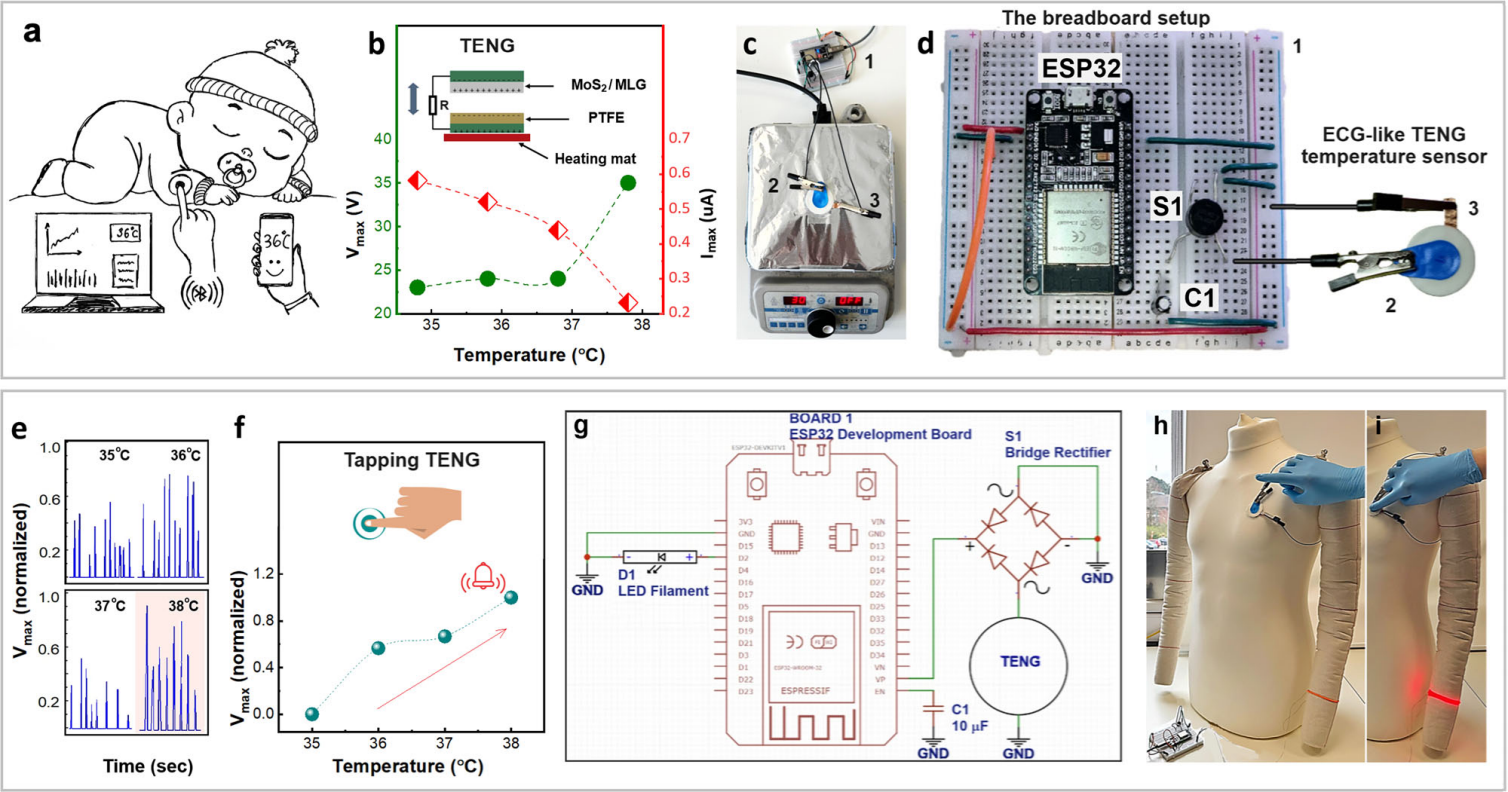
MoS_2_-TENG tap-to-sense temperature sensor. **a** Illustration of a wearable temperature sensor for infants. **b** Schematic inset of the MoS_2_-TENG’s test in CS mode and its response to temperatures ranging from 35 to 38 °C in the TENG setup; maximum open circuit voltage (*V*_*max*_) and maximum short circuit current (*I*_*sc*_). (**c**) Experimental setup of the test in tapping mode on a hot plate, with the **d** Microcontroller-based breadboard setup used for data reading. In (**c**, **d**), 1 is the breadboard, 2 is the MLG electrode with the MoS_2_ triboelectric layer, and 3 is the Cu electrode attached to the PTFE triboelectric layer. **e** Response of the MoS_2_/MLG-TENG tapping sensor at various temperatures measured on the heat plate via the microcontroller. **f** Normalised maximum open circuit voltage of the tapping sensor in the 35–38 °C temperature range. **g** The corresponding circuit schematic for the sensing circuit integrating the bridge rectifier, ESP32 board and the flexible LED filament mounted as a bracelet on a manikin. Demonstration of the wearable temperature sensor and on the manikin: **h** the LED in the “off” state when no tap is applied; **i** the LED in the “on” state upon tapping.

The development of triboelectric-driven self-powered temperature sensors is crucial for advancing wearable technology to offer a rapid, non-invasive, and user-friendly way to trigger sensing or activation, making it highly beneficial in healthcare, such as elderly and infant care (Fig. 5a). By integrating a tap-to-sense mechanism, these sensors could enable caregivers to accurately and rapidly monitor body temperature with a simple tap, eliminating the need for continuous contact or invasive methods. This approach could allow for early detection of health issues while minimising disturbances during the monitoring process.

Leveraging the temperature sensitivity of MoS_2_^58,59^, we investigated the temperature-dependent electrical response of the MoS_2_/MLG-TENG sensor by conducting measurements of the device’s electrical output at various temperatures. The device was operated at a frequency of 1 Hz in CS mode within the TENG setup described in Section 3.6, with a heating mat integrated into the stable electrode holder to ensure consistent temperature conditions. The PTFE-containing component was selectively heated from ~35 to 38 °C to simulate body temperature variations (Fig. 5b, inset). The MoS_2_/MLG-TENG sensor demonstrates an increase in voltage output and a decrease in current output, as shown in Fig. 5b, with the maximum voltage reaching ~30 V and the lowest current at ~200 nA, observed at 38 °C, with corresponding responsivities of *R*_*v*_ = 59% and *R*_*I*_ = 66%. A similar relationship was observed for the maximum charge output (Fig. S21). These trends highlight the sensor’s temperature sensitivity and its potential for detecting physiologically relevant temperature variations.

To rationalise this temperature-dependent behaviour, the physical mechanisms governing triboelectric charge generation and transport must be considered. Temperature fundamentally modulates triboelectric charge generation and charge transport in TENG systems, providing a physical basis for the observed behaviour. Increasing temperature reduces the electrical output by decreasing the dielectric’s ability to retain charges and facilitating the escape of trapped electrons^60^. In our MoS₂/MLG‑TENG, elevated temperature increases electron mobility and thermionic emission at the triboelectric interface, altering surface charge density and charge transfer efficiency, and thereby modulating voltage and current outputs. In particular, the semiconducting MoS₂ layer exhibits a high temperature coefficient of resistance, where small temperature changes rapidly modulate conductance by enhancing carrier mobility and altering charge transport pathways^58^. Additionally, temperature-induced structural disorder at the MoS₂−PTFE interface can alert the electron transfer rate between interacting triboelectric layers, effectively changing the local dielectric environment and enhancing the temperature response^60^. However, surface perturbations caused by defects, functionalisation, and environmental humidity can facilitate backward electron transfer and increase charge dissipation, ultimately reducing output performance.

It is worth noting that the temperature responsivity differs significantly from both the humidity response and the VOC signatures. This distinctive responsivity profile further reinforces the sensor’s multifunctionality and its ability to decouple different stimuli based on their unique electrical signatures. The relatively high and well-balanced voltage and current responsivities associated with temperature sensing reflect the thermally driven modulation of charge carrier transport across the MoS_2_ layer. These characteristics not only enable reliable detection of elevated body temperature using a self-powered actuation mode but also minimise the risk of cross-sensitivity with coexisting environmental stimuli. This decoupling is critical for real-world applications, where temperature fluctuations often accompany changes in humidity or exposure to chemical vapours, and highlights the robustness of the 2Dh-TENG platform for accurate, multiparameter sensing.

To showcase its practical applications as a wearable temperature sensor, an electrocardiography (ECG)-like MoS_2_/MLG-TENG device was fabricated following the procedures outlined in the Methods section. The device was first placed on a heating plate with the PTFE-containing component facing the plate (Fig. 5c), and its electrical performance was characterised by tapping on the device across the same temperature range (~35–38 °C) as in Fig. 5b. To capture the voltage output from the temperature-sensing MoS_2_/MLG-TENG and transmit it to a computer for analysis, a reading circuit was integrated (Fig. 5d). This circuit includes a full-bridge rectifier to remove negative signal components before feeding the processed signal to an ESP32 WROOM Development Board, housing an ESP32 microcontroller. The ESP32 board was programmed (see Supplementary Information) using the open-source Arduino Integrated Development Environment (IDE) to measure and log the TENG voltage as a function of time and display the data on the IDE serial monitor. Figure 5e presents the voltage response recorded from the serial monitor during device tapping on the heating plate at varying temperatures (35–38 °C), exhibiting a similar trend to the results shown in Fig. 5b and demonstrating a feasible and portable solution. Furthermore, by calibrating the temperature sensor’s voltage output as the difference (Δ*V*) between maximum (*V*_*max*_) and minimum (*V*_*min*_) readings (Fig. 5f), the calibrated data exhibits a similar trend in maximum voltage output (normalised data) as in Fig. 5b, with the highest electrical output detected at 38 °C. The maximum voltage output at 38 °C clearly demonstrates the temperature-selective response of the MoS_2_/MLG-TENG, highlighting its potential for detecting elevated body temperatures through simple finger-tapping activation.

A visual indicator was integrated to enhance the device’s ability to alert users to elevated body temperatures beyond the normal range. A flexible LED filament, connected to one of the ESP32’s digital output, serves as a visual aid for TENG activation, dynamically responding to voltage magnitude (Fig. 5g). This adaptable light indicator can be seamlessly incorporated into wearable technology, including garments and accessories (Fig. 5h, i). In this setup, the code (see Supporting Information) measures the TENG voltage, controls the LED based on voltage thresholds, and logs time and voltage via serial communication. If the voltage exceeds a predefined threshold, the LED turns “on”; otherwise, it remains “off”. This process demonstrates how elevated voltage levels correspond to specific conditions —such as temperature changes—triggering a visual alert.

## Discussion

We demonstrate a multifunctional, self-powered textile-integrated TENG platform based on solution-processed MoS_2_/MLG heterostructures, establishing a new benchmark in wearable energy-autonomous sensing. Fabricated via ultrasonic spray-coating onto textile substrates using water-based dispersions of MLG and TMDs (MoS_2_, WS_2_, MoSe_2_), the lightweight (~1 g/device) system combines high power output (*V*_*oc*_ = 58 V, *I*_*sc*_ = 0.6 μA, *P*_*d*_ = 793 mW m⁻²) with mechanical flexibility, long-term durability, and robust sensing capabilities. The MoS_2_/MLG-TENG exhibits stable operation over 80 days and after repeated mechanical deformation, maintaining self-restoring performance under humid conditions. Critically, it enables immediate, tap-to-sense detection of multiple environmental and physiological stimuli, including humidity, body temperature, and volatile organic compounds, through direct modulation of triboelectric output. Notably, the device achieves record-breaking responsivity for styrene vapours (126% current responsivity), marking the first demonstration of a wearable, self-powered sensor for this clinically relevant analyte. Functionalisation of the MoS_2_ triboelectric layer with polythiophene nanoparticles further amplifies its selectivity and signal amplification, particularly for styrene, distinguishing it from more polar analytes like ethanol and isopropanol. Moreover, the sensor’s unique temperature response, thermally driven and electronically decoupled from humidity and VOC signals, enables reliable detection of elevated body temperature with minimal cross-sensitivity, demonstrating the device’s robustness under diverse practical conditions and highlighting its potential for future human studies. This work establishes a foundational approach for engineering self-powered, textile-based sensor systems using scalable, sustainable methods and positions 2Dh-integrated TENGs as a transformative platform for next-generation wearable health diagnostics and smart textiles.

## Methods

### Materials

Expanded graphite powder (≤ 20 mm particle size) was purchased from Sigma Aldrich. Powders of MoS_2_ (≤ 2 mm particle size, 99%), and WS_2_ (≤ 2 mm particle size, 99%) were acquired from Sigma Aldrich, and MoSe_2_ (99.9%) was bought from Thermo Fisher Scientific. Isopropanol (IPA, Merck, UK) was ordered from Merck UK, and polytetrafluorethylene film (PTFE, 0.25 mm thick, 600 mm width, 1000 mm length) was purchased from Avantor. White polyester (M-09 305-A01) textile was provided by Heathcoat Fabrics Ltd. The following electronic materials were purchased from RS Components: copper double-sided tape (3 M 1182 conductive metallic tape, 25 mm × 16 m), copper wire (RS PRO single core 0.5 mm diameter copper wire, 286 m long), polyimide film (Kapton HN thermal insulating film, 304 mm × 200 mm × 0.075 mm), polyimide tape (3 M Scotch 1205 amber polyimide film electrical tape, 12 mm × 33 m), and conductive silver paste (MG Chemicals 8331S liquid adhesive, 15 g). ESP32 DEVKITV1 Microcontroller was purchased from Amazon UK, and the red 130 mm flexible LED filament was purchased from AliExpress.

### Liquid-phase exfoliation of 2D materials

The high-shear exfoliation of *graphene* from its bulk form, expanded graphite (1 g), was performed in a mixture of deionised water (DI) and IPA with a ratio of 3:1 (600 ml of DI and 200 ml of IPA). We utilised the Silverson lab mixer (L5SU model) at a rotor speed of 7000 rpm for 120 min. After allowing 30 min. For precipitation to remove any unexfoliated product, a graphene dispersion with a concentration of 1.5 g/L was achieved.

*Transition metal dichalcogenides*, MoS_2_ (1 g), MoSe_2_ (1 g) and WS_2_ (1 g), were exfoliated from their bulk forms using the same conditions as for graphene. The process was carried out for 120 min at a rotor speed of 7000 rpm. The solvent used was a mixture of DI and IPA in a volume ratio of 3:1. The concentration of the as-prepared dispersions was as follows: 1.9 g L^−1^ for MoS_2_, 1.2 g L^−1^ for MoSe_2_, and 1.7 g L^−1^ for WS_2_.

### Spray coating of 2D materials

The *deposition of exfoliated 2D materials* on textiles was carried out on a SONO-TEK ExactaCoat spray coater assembled with an ultrasonic nozzle (48 kHz); the system is capable of large area coatings up to 400 mm × 400 mm. The dispersion of 2D materials was loaded in a syringe assisted with ultrasound to prevent precipitation of the particles in the solvent and achieve a uniform coating of 2D materials on the textile. The deposition procedure was performed at 6.5 bar air pressure with a flow rate of 0.7 mL min^−1^. The textile substrate was placed on a hot plate preheated at 120 °C to quickly evaporate the solvent. The number of cycles, which represents the number of passes over the textile surface, was 200 for the first graphene coating (electrode layer), followed by 100 passes for the second TMD coating (triboelectric layer).

### TENG design and device fabrication

A sandwich-like small area (1.5 cm × 2.5 cm) double electrode TENG integrated with 2D heterostructures on a textile consists of two components. *Component I* comprises a polyester textile substrate, a spray-coated graphene layer, and a spray-coated TMD (MoS_2_, MoSe_2_, WS_2_) layer. In this configuration, graphene functions as the electrode material, while TMD serves as the triboelectric layer. *Component II* of the device is fabricated using polyester textile, a double-sided sticky Cu electrode, and on top of it is affixed a triboelectric layer made from PTFE.

### Characterisation methods

*Dynamic laser scattering* (DLS; ZetasizerZS90, Malvern Instruments) was utilised to assess the 2D materials’ particle size distribution following exfoliation in the solvent as a function of intensity. The measurements were performed in a glass cuvette, and the results represent average data derived from the three consecutive runs.

*X-ray powder diffraction* (XRD) was employed to determine the crystallinity and structural evolution of the bulk and exfoliated samples. The XRD data were collected at room temperature on a Bruker D8 powder diffractometer with parafocusing Bragg–Brentano geometry using CuKα radiation (*λ* = 0.15418 nm, *U* = 40 kV, *I* = 40 mA). The data was scanned over the angular range of 5–90° (2θ) with a step size of 0.02° (2θ).

*Raman spectroscopy* was used (Renishaw InVia Quantor) to analyse the 2D materials structure. The spectrometer was assembled with an inVia Raman microscope. The samples were transferred from the solutions onto a PTFE filter membrane by vacuum filtration and dried at ambient temperature for 24 h. They were then placed on a glass microscope slide and positioned under the objective for focusing. Single Raman measurements were performed for each scan using a 532 nm laser excitation at 10% power and a 50× objective lens.

*Atomic force microscopy* (AFM; NT-MDT Spectrum Instruments) was employed to analyse the morphology of exfoliated 2D material flakes. The measurements were carried out under ambient conditions with a scan rate of 1 Hz and a scan line of 512. The cantilever was working in a tapping mode with a strain constant of 1.5 kN m^−1^ equipped with a standard silicon tip with a curvature radius of less than 10 nm. Samples were prepared following centrifugation at 1000 rpm for 5 min and collecting the supernatant. The supernatant was then drop-casted on a silicon wafer and dried in an oven (10–15 min at 60 °C).

*Scanning transmission electron microscopy* (STEM; Jeol 7600 F SEM) was performed for the morphological characteristics of the 2D materials. The exfoliated MLG and TMDs were deposited on the double-sided carbon tape for SEM analysis and drop-casted on the Cu-grid with the holey carbon for the STEM tests.

*Scanning electron microscopy* (SEM) was utilised for morphological analysis of the deposited 2D materials on the textile substrate. It was performed on the TESCAN VEGA3 SEM equipped with both a secondary electron detector and a backscattered electron detector. Additionally, its Oxford Instrument X-MAXN EDS detector was utilised for chemical analysis of the samples.

*A 3D light digital microscope* (VHX-7000 series, 4 K high accuracy) was applied to analyse the thickness of the sprayed materials on a textile substrate by evaluating the 2D and 3D images of the studied structures.

### Electrical measurements

The *triboelectric characterisation* setup has a two-electrode configuration implemented in a vertical CS mode. In this design, two triboelectric materials (TMD vs PTFE) were positioned horizontally, facing each other. The characterisation setup for the TENG devices is performed using a motion controller. A linear motor incorporating a voice coil actuator is linked to an aluminium moving stage. The setup comprises a sample stage on the left, isolated from the rest of the moving stage by a polymer sheet. On the opposite side, the stationary sample holder remains insulated from the load cell. This load cell serves the purpose of assessing and computing the applied force, consistently maintained at approximately 40 N. The pre-cycling step to remove the pre-existing charges from triboelectric materials was purposely avoided, as the device was aimed at real-life scenarios without any primary optimisation. The characterisation parameters encompassed open circuit voltage (*V*_*oc*_), short circuit current (*I*_*sc*_), and open circuit charge (*Q*_*oc*_), all measured using a Keithley 6514 instrument. Employing the linear motor, a sinusoidal waveform was employed to facilitate the contact separations. This enabled the acquisition of alternating current (AC) signals for each parameter via electrical leads and wires. The peak values recorded during these measurements were subsequently selected for analysis. The stability tests of fabricated TENGs were carried out by assessing their performance after specific time intervals of 5, 10, and 180 days.

*Humidity test* was performed to study the stability of the proposed triboelectric materials and corresponding devices under different humidity conditions. The electrical output performance of the fabricated TENGs was examined by exposing them to relative humidity (RH) levels ranging from 20% to 70%. For this purpose, the fabricated TENGs were placed within a custom-made plastic glove box, equipped with both a humidifier and a humidity meter, enabling precise control and measurement of humidity. Controlled humidity levels were attained using a controlled flow of nitrogen gas. The triboelectric behaviour of the devices was evaluated through cyclic physical stimulation at a frequency of 1 Hz, carried out across various humidity levels.

*Bending test* was conducted to demonstrate the flexibility and mechanical robustness of the fabricated TENGs. *Component I* of the device, which consisted of TMD-MLG on polyester, underwent a series of bending tests spanning 200 cycles. *Component I* was manually bent back and forth at predetermined times, including 5, 10, 35, 50, 70, 100, 150 and 200 cycles. The effect of the bending cycle number on the TENGs’ electrical performance was analysed after each bending cycle and compared with the device’s performance prior to bending.

### Characterisation of MoS_2_/MLG-TENG-enabled wearable sensors

The MoS_2_/MLG-TENG-enabled *vapour sensor* was assembled into a TENG setup and isolated within a plastic chamber to precisely control the vapour environment to which the device would be exposed. The organic vapour environment was created using concentrated solutions of VOC placed into the plastic chamber. Vapour-sensing triboelectric behaviour of the sensor was evaluated through cyclic physical stimulation at a frequency of 1 Hz, carried out under direct exposure to ethanol, isopropanol, acetone, hexane, toluene, and styrene. The measurement under ambient conditions was used as a reference.

The MoS_2_/MLG-TENG-enabled *styrene sensor* was fabricated as detailed in Section 3.4. The sensing triboelectric MoS_2_ layer was functionalised with polythiophene nanoparticles (PTh-NPs) as biomarkers for styrene detection. 1 to 6 droplets of 5 µL each of PTh-NPs were drop-casted onto the MoS_2_ layer. Following PTh-NPs deposition, the sample was dried on a hotplate for 10−15 min. at 70 °C. The functionalised device, f-MoS_2_/MLG-TENG, was then placed in the TENG setup and isolated within a plastic chamber containing styrene vapours (100% solution). The triboelectric-driven biosensing properties of f-MoS_2_/MLG-TENG were measured at a frequency of 1 Hz and under ambient conditions. Measurements under normal air conditions were used as a reference.

The MoS_2_/MLG-TENG-enabled *temperature sensor* was designed following the methodology outlined in Section 3.4, with two configurations. Firstly, for direct measurements within the TENG setup in CS mode, and secondly, to evaluate the device on a heat plate in tapping mode.

In the first configuration, the device was integrated into the TENG setup, with *Component I* attached to the moving head and *Component II* to the heating mat (Seedling Heat Mat with Dual Display Digital Thermostat, from amazon.co.uk), affixed to the stable electrode holder. The temperature of the heating mat was manually adjusted to simulate human body temperatures ranging from 35 to 38 °C, and the device’s electrical output was measured at a frequency of 1 Hz.

For the second configuration, a wearable sensor akin to an attachable ECG electrode was fabricated. The device was positioned in direct contact with the PTFE-containing element on the heat plate, and its electrical output was measured in tapping mode while subjecting the device to varying temperatures between 35 and 38 °C.

To facilitate the measurement of the voltage output from the *tapping-driven MoS*_*2*_*/MLG-TENG temperature sensor* and transmit it to a computer for analysis, a reading circuit was integrated with the sensor, comprising an ESP32 WROOM Development Board, housing an ESP32 microcontroller with a 2.4 GHz clock speed and a 12-bit analogue-to-digital converter (ADC). This setup enables data acquisition, with the potential for future integration into remote monitoring^61^ systems via Wi-Fi and Bluetooth Low Energy (BLE 4.0). To ensure accurate signal processing, a full-bridge rectifier was incorporated to eliminate negative signal components before feeding the signal into the ESP32’s GPIO pin for ADC conversion^62^. The ESP32 board was programmed using the open-source Arduino Integrated Development Environment (IDE)^63^. The code (see Supporting Information) periodically measures the TENG voltage at ESP32’s pin 36, and logs time and voltage via serial communication. The print voltage function converts the raw ADC value to a voltage by considering the ESP32’s maximum ADC range and resolution, displaying this voltage on the serial monitor with two decimal precision for better readability and ease of data processing. An open-source serial oscilloscope software was employed to visualise voltage data, utilising comma-separated serial data from the microcontroller and plotting it in real time^64,65^. This programme enabled data acquisition and exportation for analysis as a .csv file.

## Supplementary information

Supplementary information

## Data Availability

The datasets generated and/or analysed during the current study are not publicly available due to their large size, but are available from the corresponding author upon reasonable request.
