# Supplementary material for: Multifunctional, energy-autonomous textile sensors enabled by spray-coated two-dimensional heterostructures
Source: Npj Flex Electron. 2026 Feb 24;10(1):49. doi: 10.1038/s41528-026-00539-3 (PMC13043307; doi:10.1038/s41528-026-00539-3)
Supplement: Supplementary file 1 — Supplementary information [file 41528_2026_539_MOESM1_ESM.pdf]

Supporting Information

Multifunctional, energy-autonomous textile sensors enabled by spray-coated two-dimensional heterostructures

Evgeniya Kovalska,[1*] Jack Routledge,[1] Rocco Cancelliere,[2,3] Hoi Tung Lam,[2] Kavya Sreeja Sadanandan,[1] Bing Wu,[4] Liping Liao,[4] Zdenek Sofer,[4] Ana I.S. Neves,[1] Saverio Russo,[2] Laura Micheli,[3] Monica F. Craciun[1*]

[1]*Faculty of Environment, Science and Economy, Department of Engineering, University of Exeter, EX4 4QF, Exeter, United Kingdom*

[2]*Faculty of Environment, Science and Economy, Department of Physics and Astronomy, University of Exeter, EX4 4QL, Exeter, United Kingdom*

[3]*Department of Chemical Sciences and Technologies, University of Rome Tor Vergata, Via della Ricerca Scientifica 1, 00133 Roma, Italy*

[4] *Department of Inorganic Chemistry, University of Chemistry and Technology Prague, 166 28, Prague, Czechia*

Contents:

Fig. S1. DLS analysis of the average size distribution of 2D material particles in the corresponding dispersions. Measurements were performed using a glass cuvette, with the results presented as three curves corresponding to three consecutive runs. The DLS size distribution is displayed with the particle diameter (in nm) on the x-axis and the particle count (in %) on the y-axis.

Fig. S2. DLS analysis of the Multilayer Graphene (MLG) particle size distribution in the dispersion. The results of the DLS size distribution are shown with the particle diameter in nm on the x-axis and the number of particles in % on the y-axis.

Fig. S3. DLS analysis of the WS_2 particle size distribution in the dispersion. The results of the DLS size distribution are shown with the particle diameter in nm on the x-axis and the number of particles in % on the y-axis.

Fig. S4. DLS analysis of the MoS_2 particle size distribution in the dispersion. The results of the DLS size distribution are shown with the particle diameter in nm on the x-axis and the number of particles in % on the y-axis.

Fig. S5. DLS analysis of the $MoSe_2$ particle size distribution in the dispersion. The results of the DLS size distribution are shown with the particle diameter in nm on the x-axis and the number of particles in % on the y-axis.

Fig. S6. Morphological analysis of exfoliated TMD flakes: Atomic Force Microscopy (AFM) images and corresponding height profiles of (**a, b**) MoS_2, (**c, d**) WS_2 and (**e, f**) $MoSe_2$.

Fig. S7. SEM images of TMDs on MLG deposited on textile: (**a, b**) MoS_2, (**c, d**) WS_2, and (**e, f**) $MoSe_2$.

Fig. S8. 3D light microscopy of WS_2_MLG_polyester: left upper corner – 3D image; right upper corner – 2D image and profile across uncoated and coated areas of polyester; below graph – enlarged profile across the sample.

Fig. S9. 3D light microscopy of MoS_2_MLG_polyester: left upper corner – 3D image; right upper corner – 2D image and profile across uncoated and coated areas of polyester; below graph – enlarged profile across the sample.

Fig. S10. 3D light microscopy of $MoSe_2$_MLG_polyester: left upper corner – 3D image; right upper corner – 2D image and profile across uncoated and coated areas of polyester; below graph – enlarged profile across the sample.

Fig. S11. Triboelectric series of tested materials; open circuit voltage output.

Fig. S12. Electrical output of TMD-TENGs in (**a**) parallel and (**b**) concave/convex configurations.

Fig. S13. Open-circuit charge of the MoS_2-TENG **(a)** over time and (**b**) under mechanical deformations.

Fig. S14. MoS_2/MLG-TENG humidity sensor. (**a**) Open-circuit voltage output (V_{oc}) of the device showing a rapid response during a 30 s exposure to elevated humidity. (**b**) The first peak (T_1 = 12.04 s) corresponds to room humidity (~45-50% RH), while the subsequent peak (T_2 = 13.02 s) is recorded immediately after the introduction of higher humidity. (**c**) Open-circuit voltage output (V_{oc}) of the device during argon (Ag) purging, with exposure initiated at T = 18 s, illustrating rapid recovery upon humidity reduction. (**d**) The first peak (T_1 = 18.38 s) corresponds to increased humidity (~65-70% RH), while the subsequent peak (T_2 = 19.36 s) is recorded immediately after the introduction of argon. The apparent response and recovery times are defined as the time difference between consecutive voltage peaks and are identical ($\Delta T \approx$ 0.98 s). The shaded regions indicate the exposure windows, with blue corresponding to elevated humidity and red to argon.

Fig. S15. Commercial sensor response vs MoS_2/MLG-TENG sensor. Relative humidity (RH) measured using a commercial sensor (GoveeLife Thermometer Hygrometer) during controlled environmental modulation. Shaded regions indicate time intervals for elevated humidity exposure and argon purging.

The humidity changes of commercial sensor (black curve profile) align with the changes of the MoS_2/MLG-TENG open circuit voltage (V_{oc}) profile.

Fig. S16. Humidity sensing resolution. **(a)** Continuous-mode operation of the TENG sensor showing voltage peak shifts in response to changes in humidity, with calibrated relative humidity (RH) values provided by a commercial reference sensor. **(b)** Correlated evolution of the maximum open-circuit voltage, V_{oc} and relative humidity, showing a monotonic decrease of V_{oc} with decreasing RH. Based on the minimum resolvable voltage fluctuation (~0.05 V), the humidity sensing resolution is estimated to be on the order of 0.1% RH under dynamic sensing conditions.

Fig. S17. Open circuit charge, Q_{oc} output of the MoS_2-TENG under exposure to various volatile organic compounds (air, ethanol, propanol, acetone, heptane, toluene and styrene).

Fig. S18. Open circuit charge, Q_{oc} output of the MoS_2-TENG under exposure to various volatile organic compounds (air, ethanol, propanol, acetone, heptane, toluene and styrene).

Fig. S19. Open circuit charge, Q_{oc}. of bare and functionalised MoS_2-TENG.

Fig. S20. Open circuit charge, Q_{oc} output of the MoS_2-TENG under exposure to styrene as a function of nanoparticles concentration.

Fig. S21. Open circuit charge, Q_{oc}. of MoS_2-TENG sensor under various temperature conditions.

The Arduino code

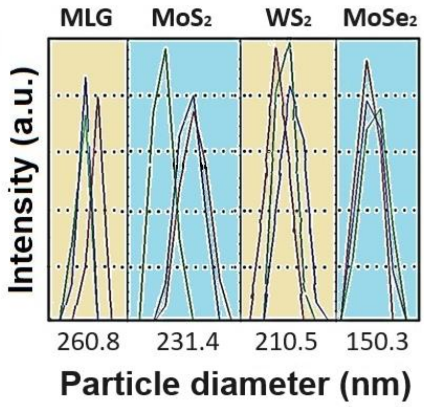

Fig. S1. DLS analysis of the average size distribution of 2D material particles in the corresponding dispersions. Measurements were performed using a glass cuvette, with the results presented as three curves corresponding to three consecutive runs. The DLS size distribution is displayed with the particle diameter (in nm) on the x-axis and the particle count (in %) on the y-axis.

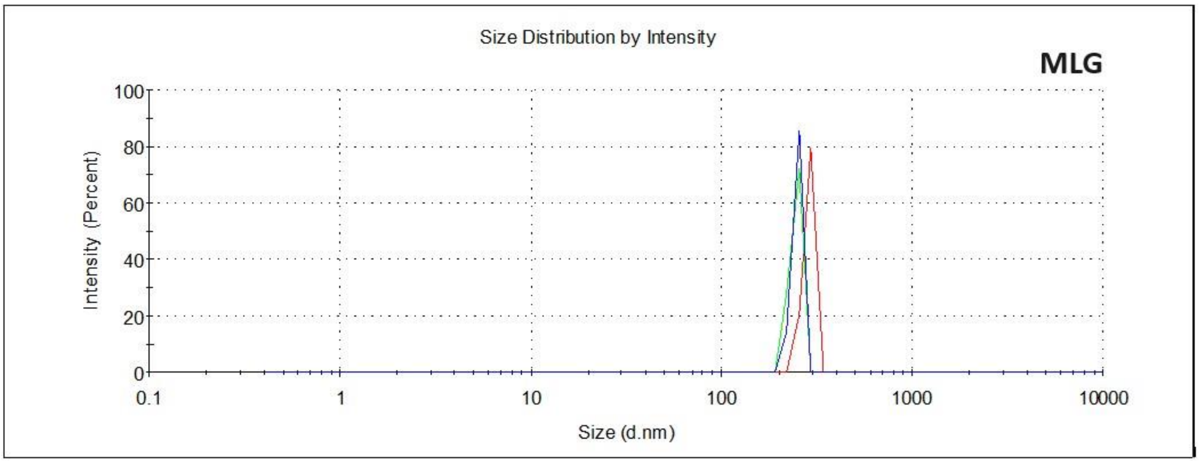

Fig. S2. DLS analysis of the MLG particle size distribution in the dispersion. The results of the DLS size distribution are shown with the particle diameter in nm on the x-axis and the number of particles in % on the y-axis.

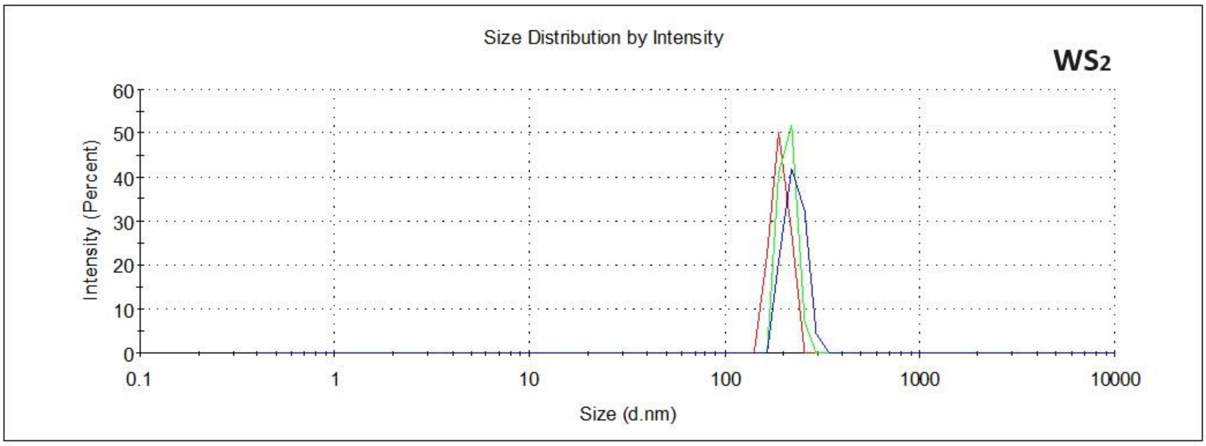

Fig. S3. DLS analysis of the WS_2 particle size distribution in the dispersion. The results of the DLS size distribution are shown with the particle diameter in nm on the x-axis and the number of particles in % on the y-axis.

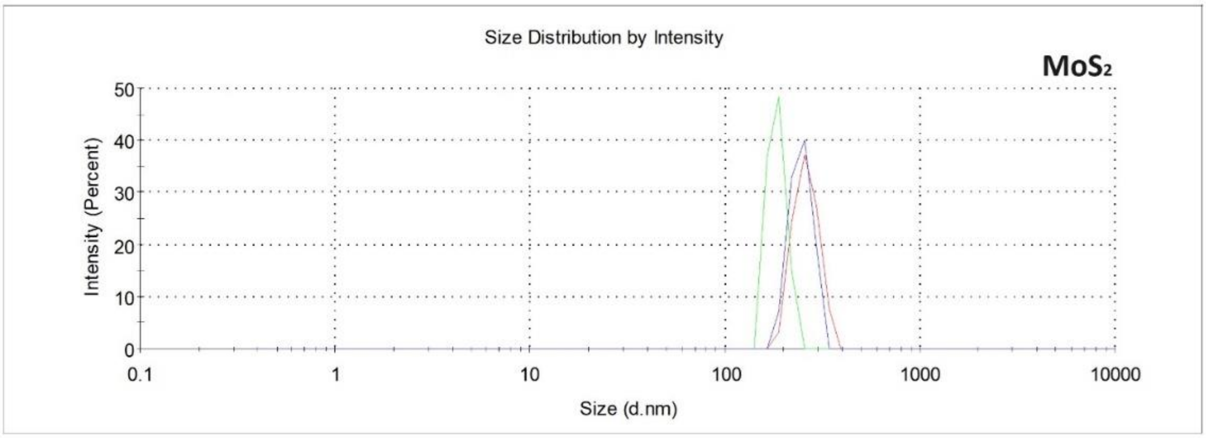

Fig. S4. DLS analysis of the MoS_2 particle size distribution in the dispersion. The results of the DLS size distribution are shown with the particle diameter in nm on the x-axis and the number of particles in % on the y-axis.

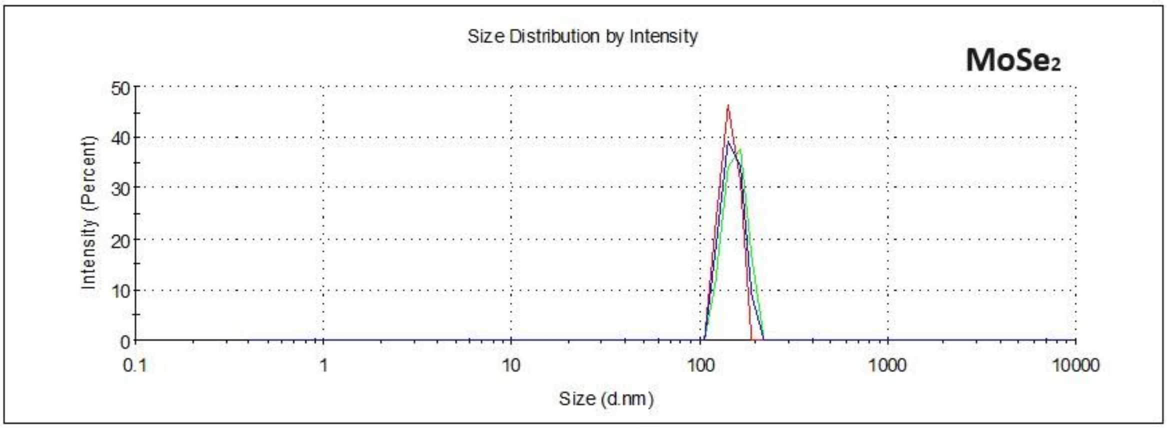

Fig. S5. DLS analysis of the $MoSe_2$ particle size distribution in the dispersion. The results of the DLS size distribution are shown with the particle diameter in nm on the x-axis and the number of particles in % on the y-axis.

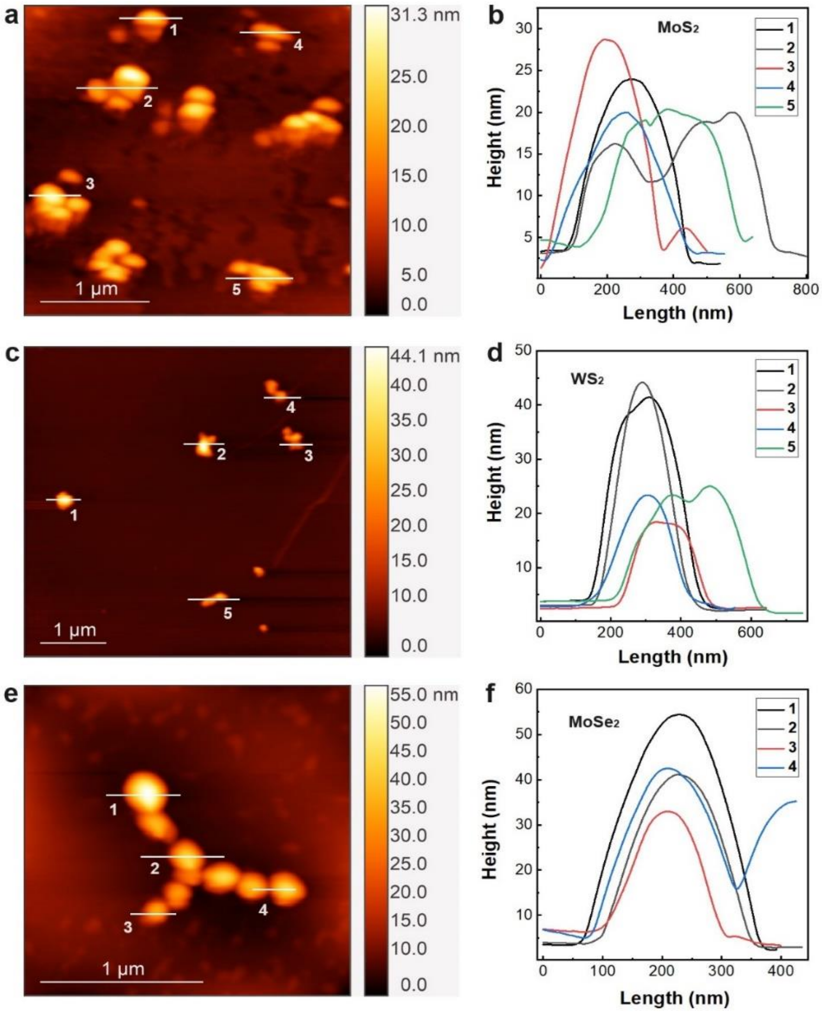

Fig. S6. Morphological analysis of exfoliated TMD flakes: AFM images and corresponding height profiles of (**a, b**) MoS_2, (**c, d**) WS_2 and (**e, f**) $MoSe_2$.

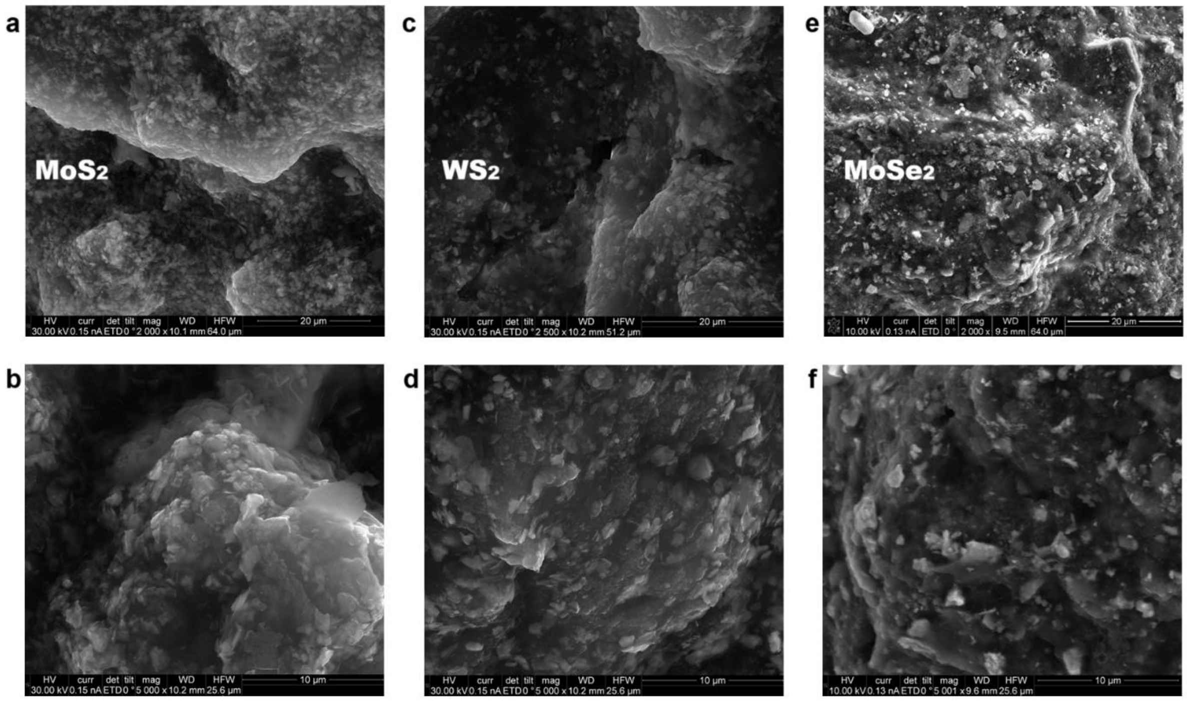

Fig. S7. SEM images of TMDs on MLG deposited on textile: (**a, b**) MoS_2, (**c, d**) WS_2, and (**e, f**) $MoSe_2$.

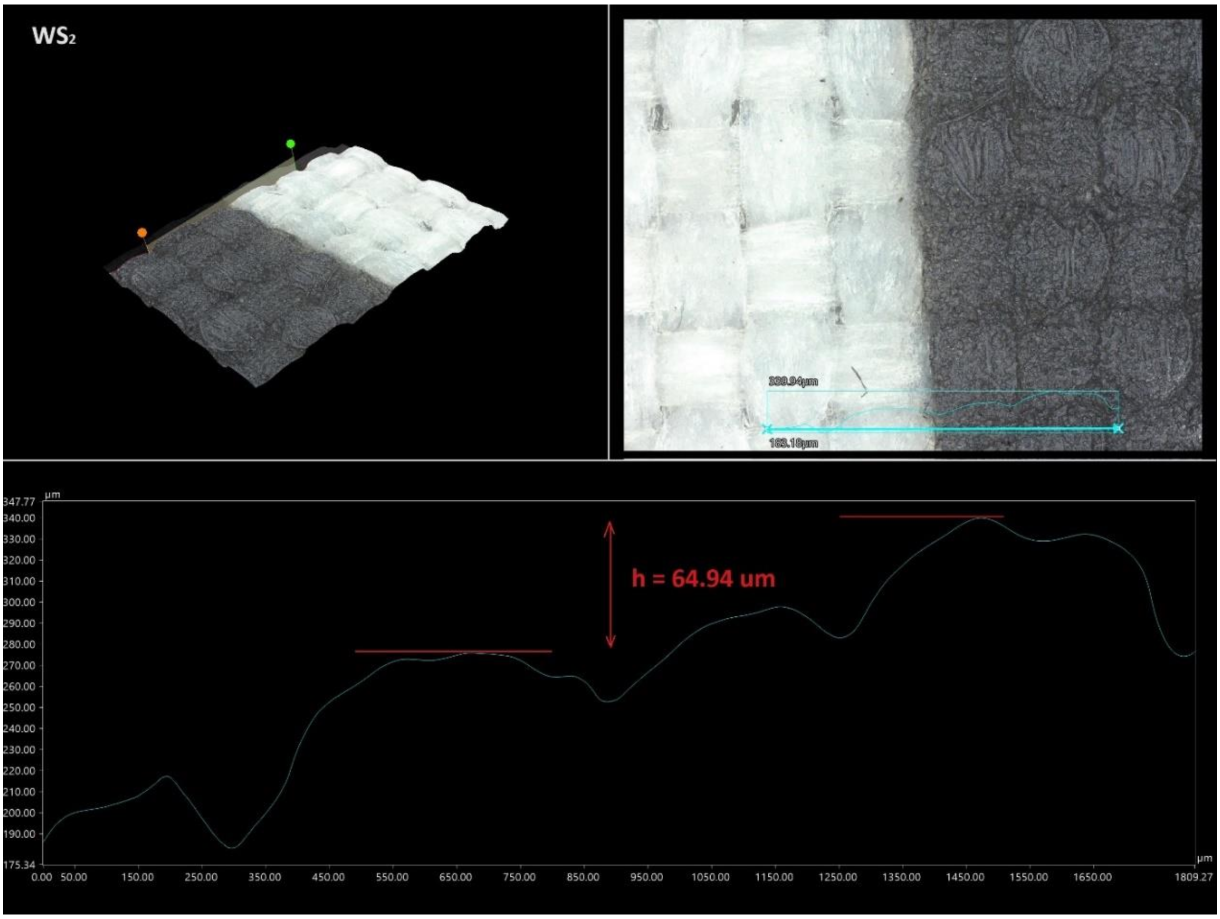

Fig. S8. 3D light microscopy of WS_2_MLG_polyester: left upper corner – 3D image; right upper corner – 2D image and profile across uncoated and coated areas of polyester; below graph – enlarged profile across the sample.

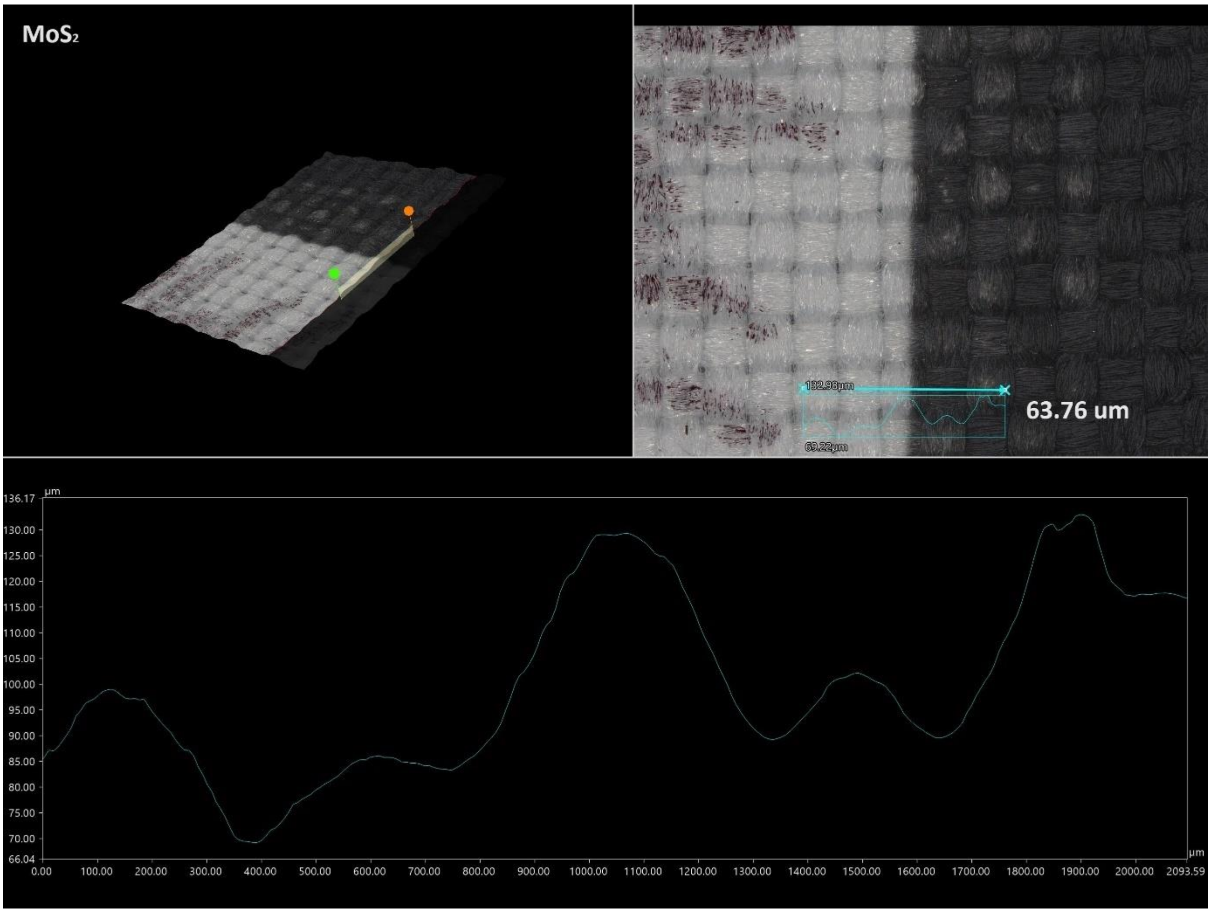

Fig. S9. 3D light microscopy of MoS_2_MLG_polyester: left upper corner – 3D image; right upper corner – 2D image and profile across uncoated and coated areas of polyester; below graph – enlarged profile across the sample.

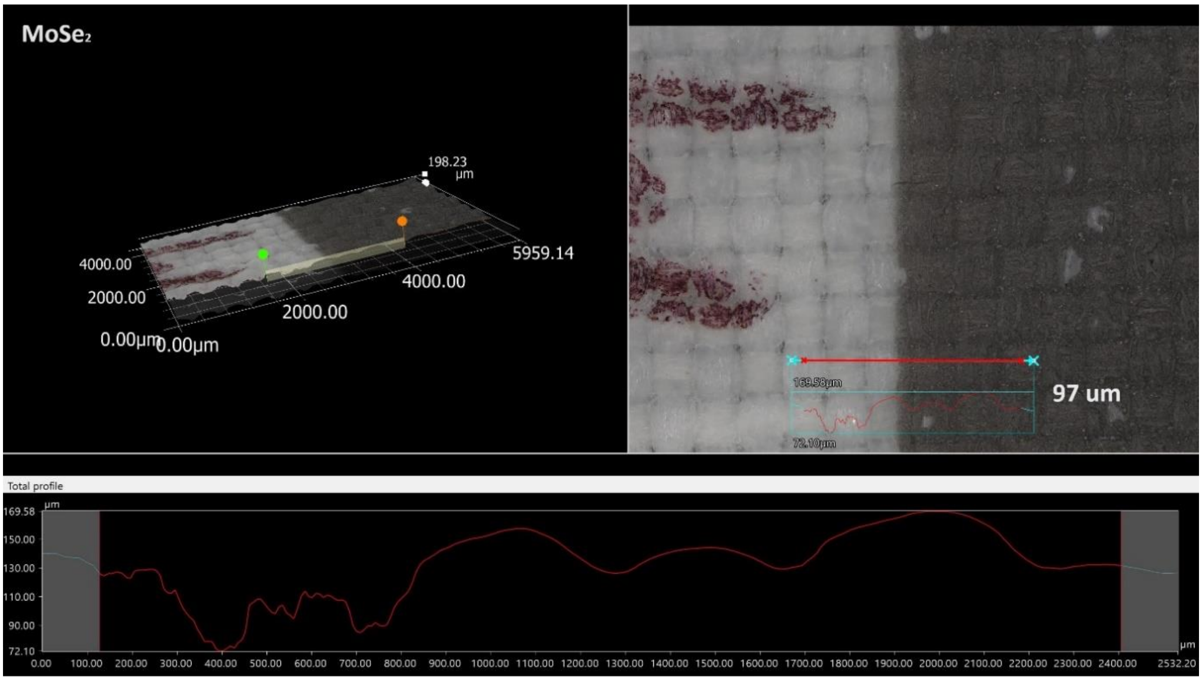

Figure S10. 3D light microscopy of $MoSe_2$_MLG_polyester: left upper corner – 3D image; right upper corner – 2D image and profile across uncoated and coated areas of polyester; below graph – enlarged profile across the sample.

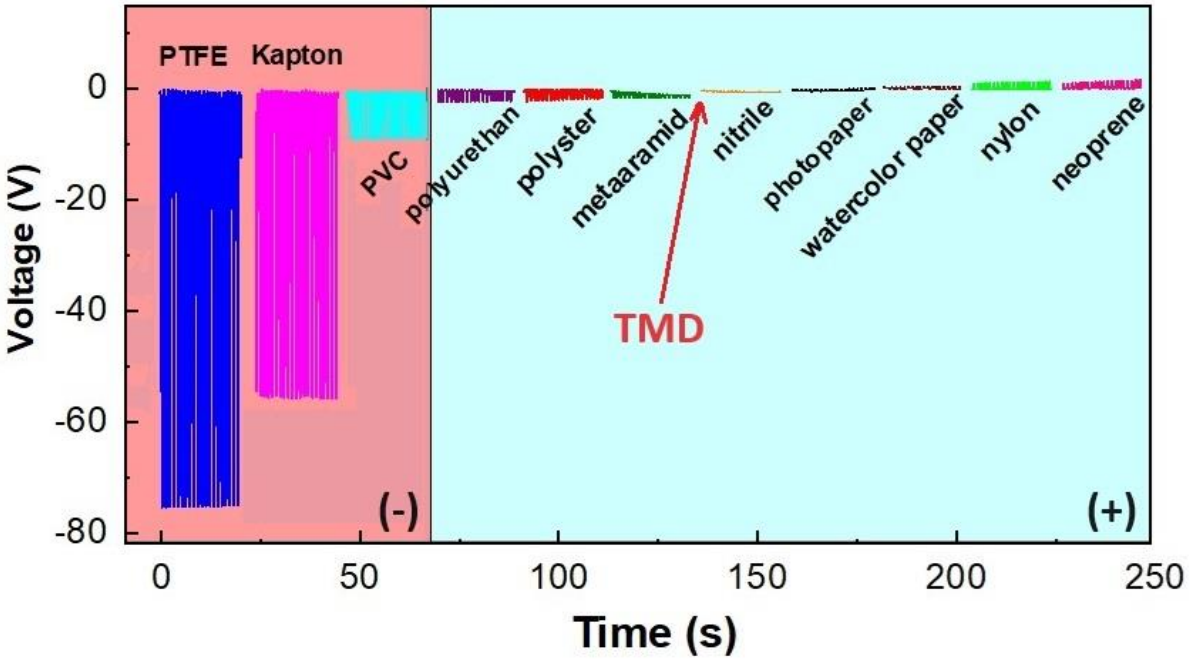

Fig. S11. Triboelectric series of tested materials; open circuit voltage output.

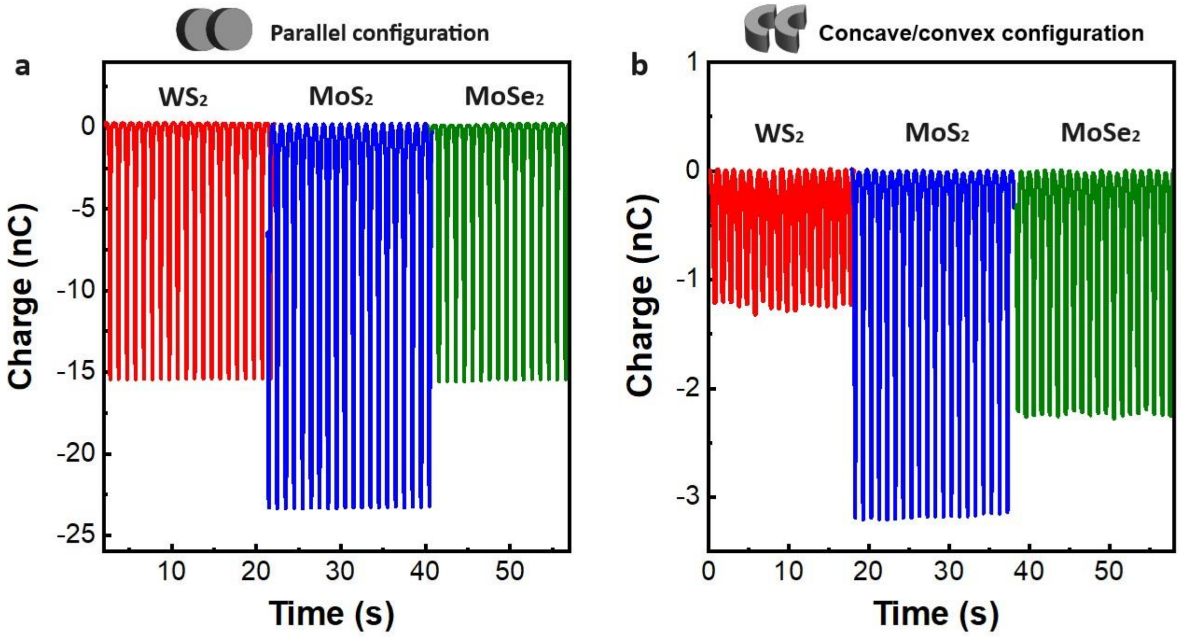

Fig. S12. Electrical output of TMD-TENGs in (**a**) parallel and (**b**) concave/convex configurations.

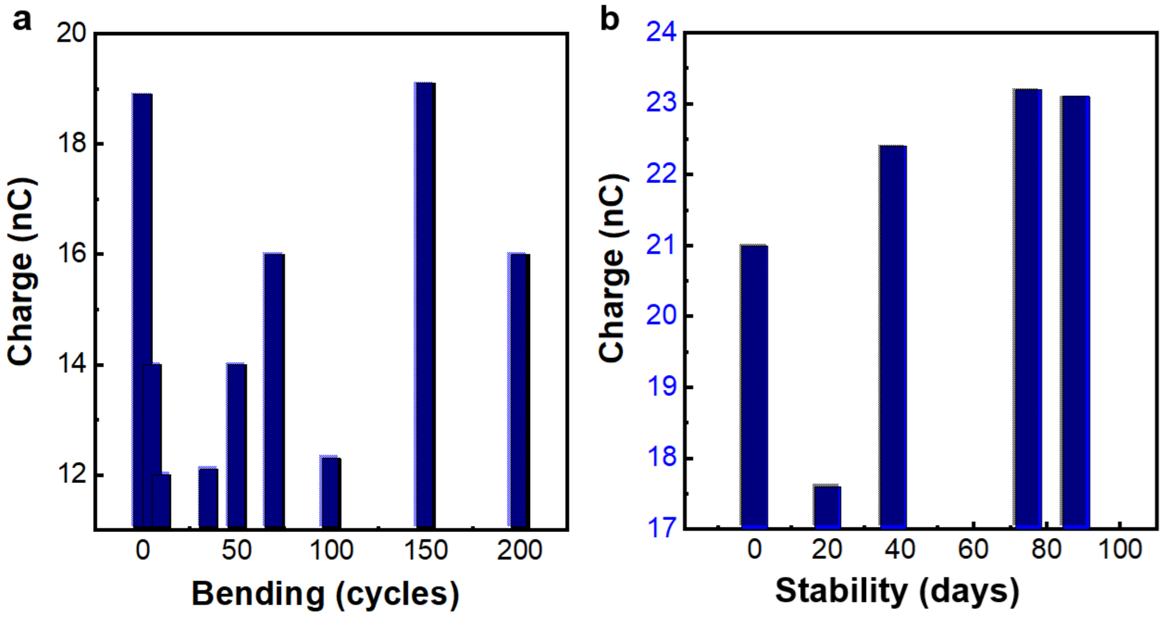

Fig. S13. Open-circuit charge of the MoS_2-TENG **(a)** under mechanical deformations and (**b**) over time.

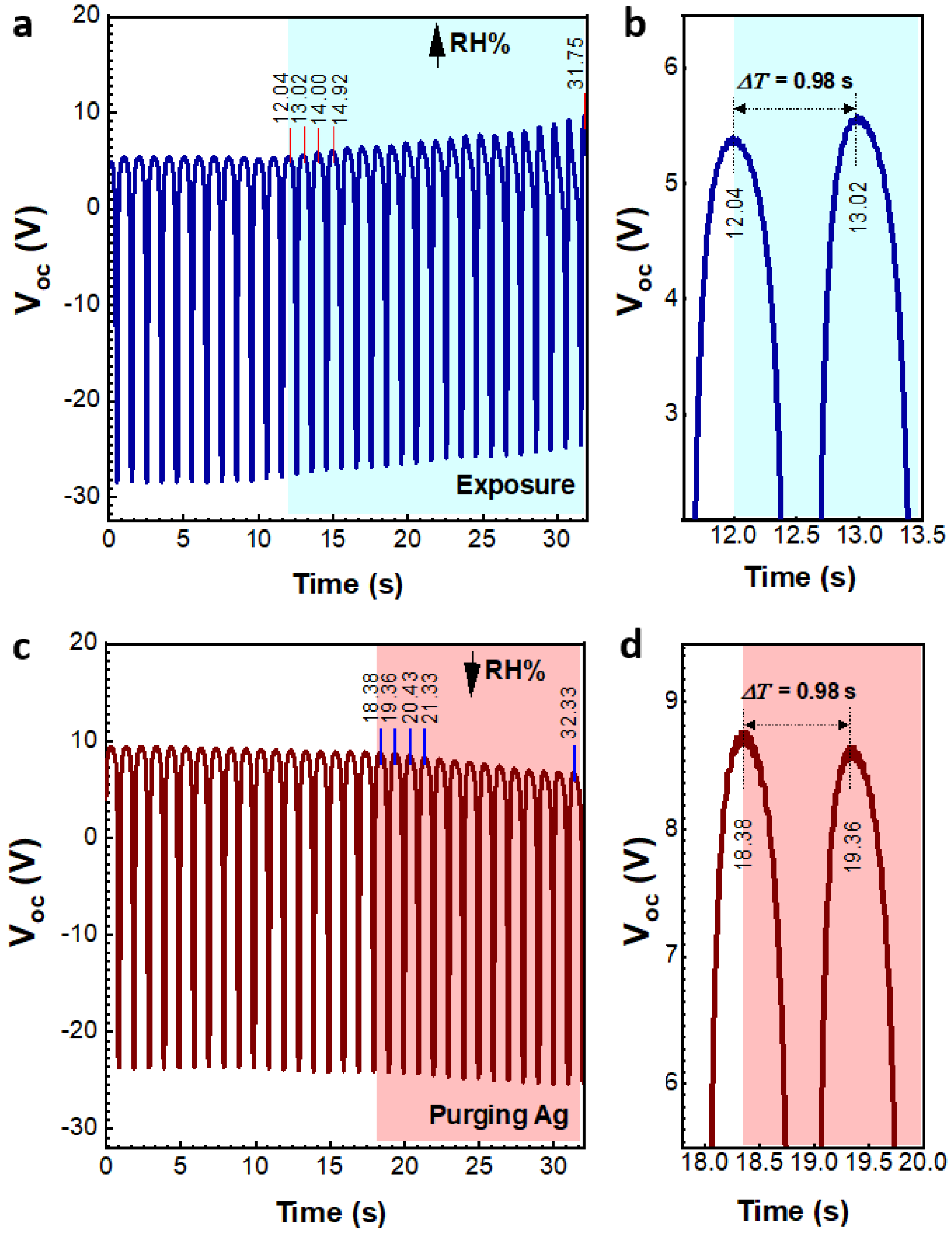

Fig. S14. MoS_2/MLG-TENG humidity sensor. (**a**) Open-circuit voltage output (V_{oc}) of the device showing a rapid response during a 30 s exposure to elevated humidity. (**b**) The first peak (T_1 = 12.04 s) corresponds to room humidity (~45-50% RH), while the subsequent peak (T_2 = 13.02 s) is recorded immediately after the introduction of higher humidity. (**c**) Open-circuit voltage output (V_{oc}) of the device during argon (Ag) purging, with exposure initiated at T = 18 s, illustrating rapid recovery upon humidity reduction. (**d**) The first peak (T_1 = 18.38 s) corresponds to increased humidity (~65-70% RH), while the subsequent peak (T_2 = 19.36 s) is recorded immediately after the introduction of argon. The apparent response and recovery times are defined as the time difference between consecutive voltage peaks and are identical ($\Delta T \approx 0.98$ s). The shaded regions indicate the exposure windows, with blue corresponding to elevated humidity and red to argon.

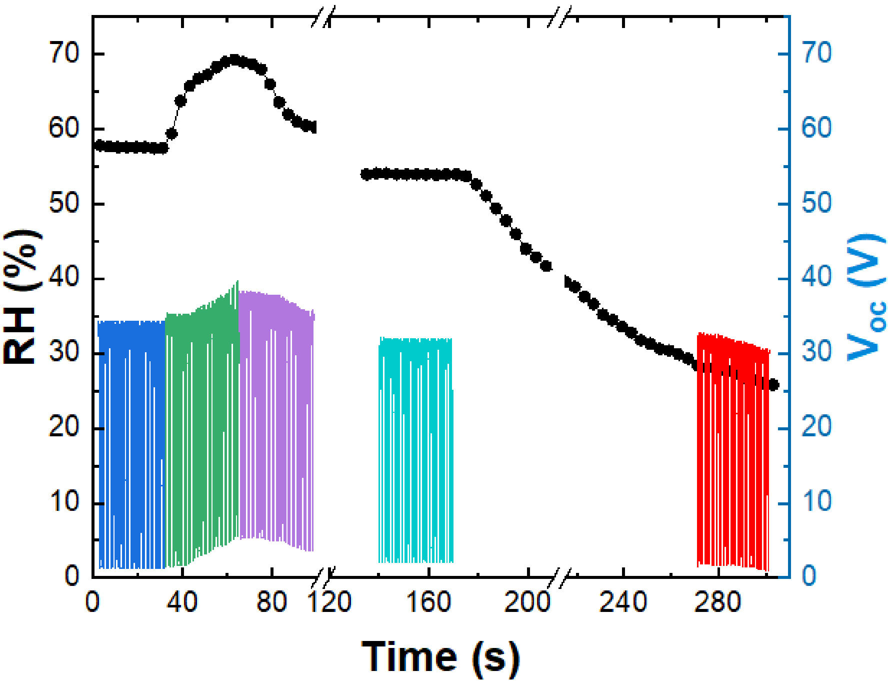

Fig. S15. Commercial sensor response vs MoS_2/MLG-TENG sensor. Relative humidity (RH) measured using a commercial sensor (GoveeLife Thermometer Hygrometer) during controlled environmental modulation. Shaded regions indicate time intervals for elevated humidity exposure and argon purging. The humidity changes of commercial sensor (black curve profile) align with the changes of the MoS_2/MLG-TENG open circuit voltage (V_{oc}) profile.

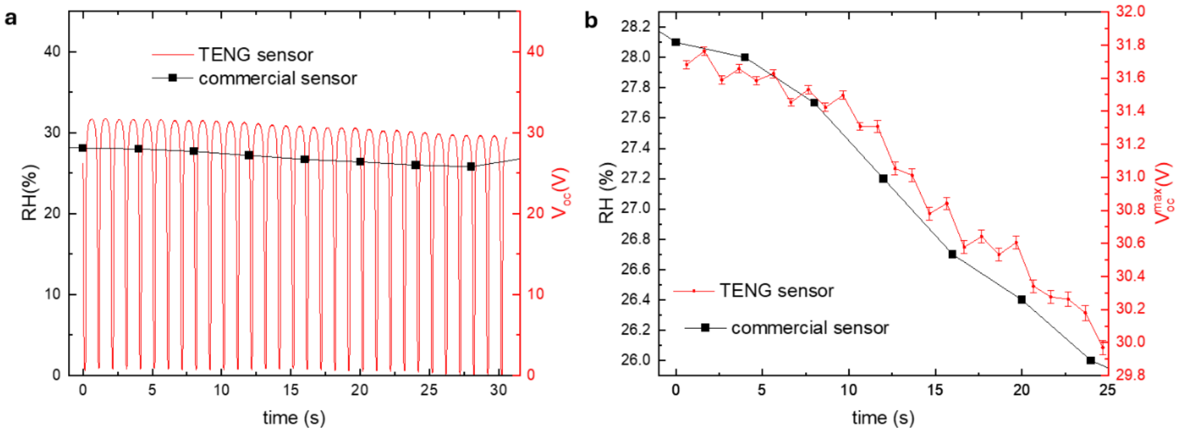

Fig. S16. Humidity sensing resolution. **(a)** Continuous-mode operation of the TENG sensor showing voltage peak shifts in response to changes in humidity, with calibrated relative humidity (RH) values provided by a commercial reference sensor. **(b)** Correlated evolution of the maximum open-circuit voltage, V_{oc} and relative humidity, showing a monotonic decrease of V_{oc} with decreasing RH. Based on the minimum resolvable voltage fluctuation (~0.05 V), the humidity sensing resolution is estimated to be on the order of 0.1% RH under dynamic sensing conditions.

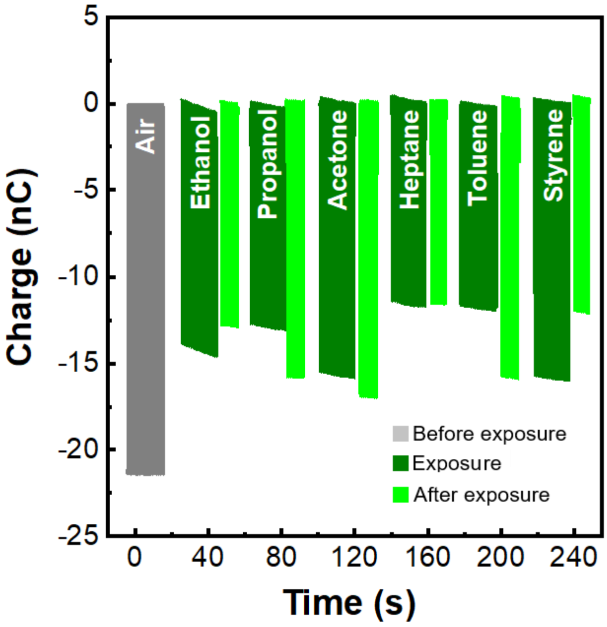

Fig. S17. Open circuit charge, Q_{oc} output of the MoS_2-TENG under exposure to various volatile organic compounds (air, ethanol, propanol, acetone, heptane, toluene and styrene).

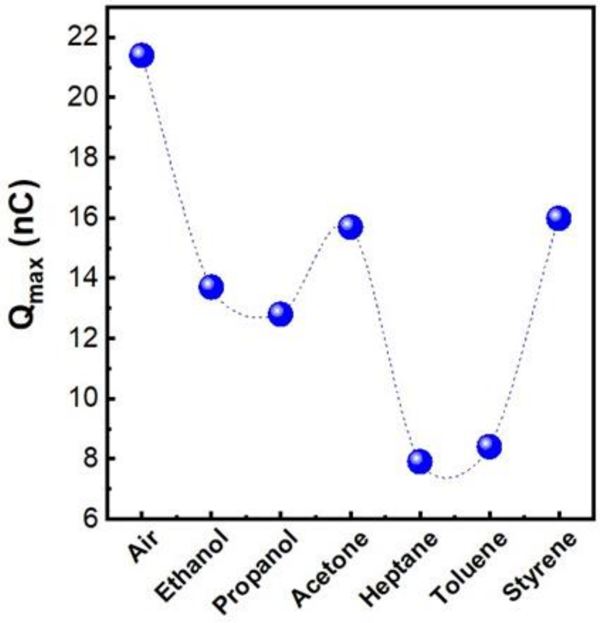

Fig. S18. Open circuit charge, Q_{oc} output of the MoS_2-TENG under exposure to various volatile organic compounds (air, ethanol, propanol, acetone, heptane, toluene and styrene).

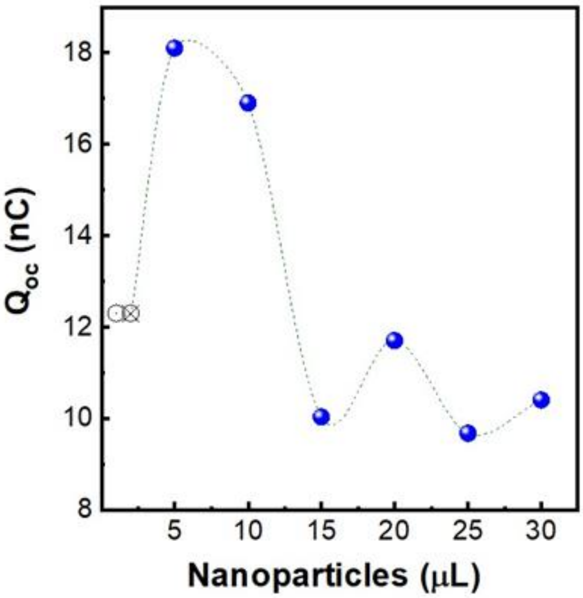

Fig. S19. Open circuit charge, Q_{oc}. of bare and functionalised MoS_2-TENG.

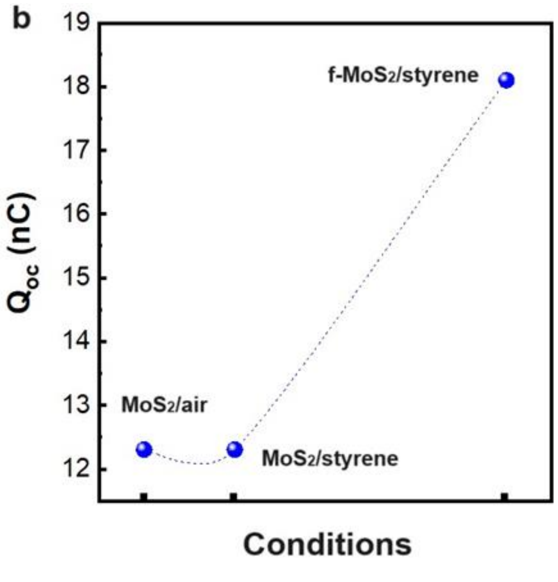

Fig. S20. Open circuit charge, Q_{oc} output of the MoS_2-TENG under exposure to styrene as a function of nanoparticles concentration.

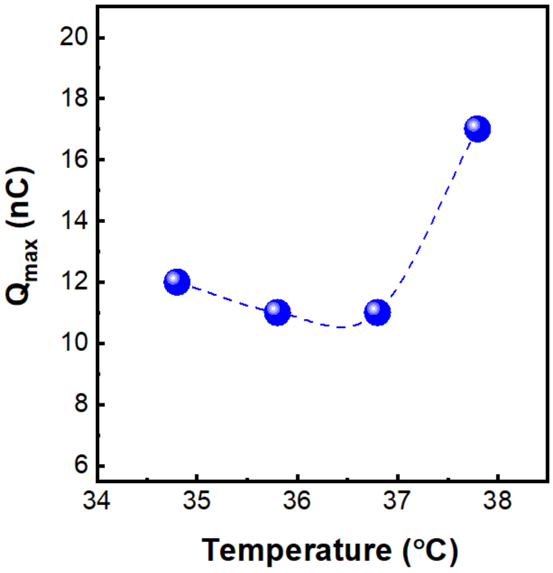

Fig. S21. Open circuit charge, Q_{oc}. of MoS_2-TENG sensor under various temperature conditions.

The Arduino code

```
#include <Arduino.h>

unsigned long previousMillis = 0;   // Store previous time when data was sent
const long interval = 100;          // Interval between data readings in milliseconds
const int outputPin = 2;            // Set Output Pin For LED Filament

void setup() {

    // Initialise serial baud rate
    Serial.begin(115200);

    // configure ADC for getting battery voltage
    pinMode(36, INPUT); // Battery Voltage Sense input
    analogSetWidth(11); // set the resolution width to be 2048 instead of the default 4096 to try and reduce noise

    // Set the output pin as an output
    pinMode(outputPin, OUTPUT);
}

void loop() {

    unsigned long currentMillis = millis(); // Get the current time

    // If it's time to send data
    if (currentMillis - previousMillis >= interval) {
        // Record the current time
        previousMillis = currentMillis;

        // Print the current time
        Serial.print(currentMillis);
        Serial.write(',');

        // Read from the specified ADC pins and print the voltage
        printVoltage(analogRead(36)); // GPIO 36 (VP)

        Serial.write('\r'); // End of line

        // Turn LED Filament ON if TENG Pressed and Above Threshold
        if(analogRead(36) > 300) {      // Adjust the threshold value as needed
        digitalWrite(outputPin, HIGH);  // Set the output HIGH
        } else {
        digitalWrite(outputPin, LOW);   // Set the output LOW
        }

    }
}

// Function to convert the ADC value to a voltage and print it:
void printVoltage(int adcValue) {
    // Convert the ADC reading to a voltage assuming a 3.3V reference:
    float voltage = adcValue * (3.3 / 4095.0);
    // Print the voltage to the serial monitor with two decimal places:
    printFloat(voltage, 2);
}

// Print a float value with specified decimal places using Serial.printf
void printFloat(float value, int places) {
    // Serial.printf allows formatted strings. %.*f specifies floating point format
    // with a dynamic precision set by 'places'. This prints 'value' with 'places'
    // number of digits after the decimal point.
    Serial.printf("%.*f", places, value);
}
```
